# A Risk-Based Pillar Design Approach Combining Stochastic Continuous and Discontinuous Modeling in an Underground Stone Mine

**DOI:** 10.1007/s42461-025-01210-7

**Published:** 2025-02-18

**Authors:** Juan J. Monsalve, Aman Soni, Richard Bishop, Jim Hazzard, Adrian Rodriguez-Marek, Cheng Chen, Nino Ripepi

**Affiliations:** 1 Mining and Minerals Engineering Department, Virginia Polytechnic Institute & State University, Blacksburg, USA; 2 ITASCA Consulting Group, Minneapolis, MN USA; 3 Civil and Environmental Engineering Department, Virginia Polytechnic Institute & State University, Blacksburg, USA; 4 Stevens Institute of Technology, Hoboken, NJ USA

**Keywords:** Pillar, Design, Risk, Stochastic, Discrete element modeling, Uncertainty, LiDAR

## Abstract

The collapse of a mine pillar is a catastrophic event with great consequences for a mining operation. In spite of the low probability of occurrence for a pillar collapse in comparison to other ground control instability issues, these consequences make these events high risk. Therefore, the design of these structures should be considered from a risk perspective rather than from a factor-of-safety deterministic approach, as it has been traditionally done. This work presents a risk-based pillar design framework that enables to characterize discontinuities’ effect in pillar strength, as well as accounting for the possible range of stresses that will be acting on pillars. The proposed methodology is based on the integration of stochastic discrete element modeling for pillar strength estimation, and stochastic continuous modeling for pillar stress determination. This approach was evaluated in an underground dipping stone mine. Using the reliability analysis method, results from the stress estimation model were integrated with those obtained from the stochastic DEM approach, thereby enabling the probability of failure estimation for the pillars throughout the mine. Finally, the methodology was validated by comparing numerical modeling results with LiDAR and photogrammetric surveys from the mine. Results from this design framework provide additional decision-making tools to prevent pillar failure from the design stages by reducing uncertainty. The proposed method enables the integration of pillar design into the risk analysis framework of the mining operation, ultimately improving safety by preventing future pillar collapses.

## Introduction

Pillar design is one of the most critical tasks during mine design for an underground room and pillar operation. The design of these support elements must ensure maximum resource recovery while maintaining long term stability of the workings. Historically, multiple pillar collapses have been reported in the US with various consequences including air blasts, cascade pillar collapses, surface subsidence, reserves sterilization, property damage, and fatalities [14, 15, 34, 37, 50]. Recent collapses highlight risks to both mine infrastructure and worker safety. From 2015 to 2022, five collapses occurred, with one causing serious injuries and the four other considered near misses. Some of these collapses have occurred in areas considered “mine legacy workings” as described by Rumbaugh et al. [39].

For many years, mine pillar design has been addressed using analytical, numerical, empirical, and observational methods, but no consensus on the best solution exists. Although pillar design traditionally relied on empirical equations derived from specific cases studies, a transition to numerical analysis, validated through observation and instrumentation, has become a more prevalent trend in the industry [20, 24, 25, 32]. Multiple researchers have shown numerical modeling techniques as a reliable tool to estimate pillar strength in underground mines [11, 12, 23, 36]. In addition, these tools have also been used to reproduce complex failure mechanisms that can occur when discontinuities are present in the rock mass, and estimate stresses around underground mine pillars under multiple design scenarios [15, 17, 31].

Probabilistic risk analysis (PRA) has become a key method for quantifying the probability of system failures by analyzing variability in design parameters. While PRA has been widely used in fields like dam safety and construction, its application to underground mining is relatively recent. Current technological advances in rock mass characterization techniques, data analysis tools, computational power, and numerical modeling including the discrete element method (DEM) and discrete fracture networks (DFNs) have boosted increased attention in probabilistic risk analysis approaches in underground mine design [18, 21, 33, 42, 45, 46]. Due to the great consequences of a pillar failure, the analysis of these events should be performed from a risk-based perspective such as PRA.

**Fig. 1:**
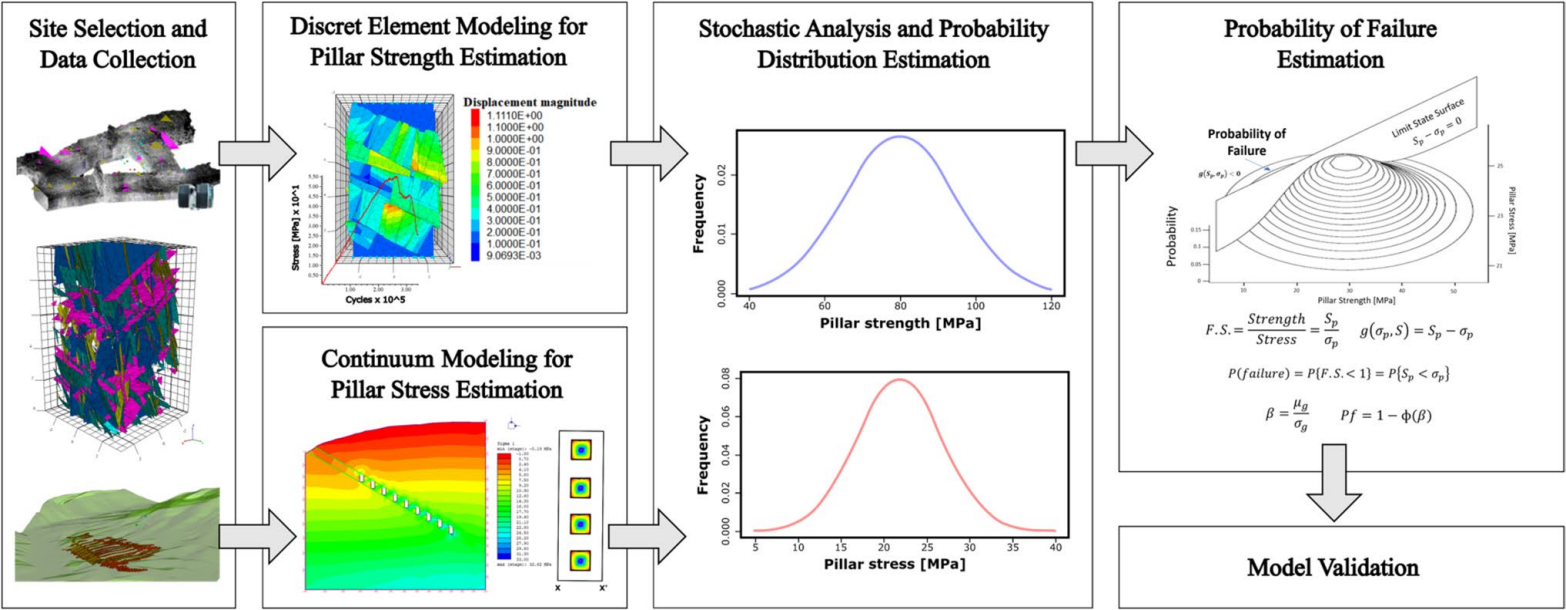
Proposed workflow for a risk-based approach for pillar design.

In previous work, the authors discussed the importance of implementing risk-based analysis methods into pillar design and proposed a risk-based approach for pillar design and stability assessment [32]. The proposed methodology is based on the integration of stochastic discrete element modeling for pillar strength estimation, and stochastic continuous modeling for pillar stress determination. The reliability method, a probabilistic risk analysis approach, was proposed to combine both results to estimate pillar probability of failure. In addition, results from numerical models used in this work are validated by comparing its results with 3D photorealisitic pillar models obtained from drone and terrestrial based LiDAR and photogrammetric surveys. Figure 1 summarizes the proposed methodology.

This paper is divided in four sections where Sect. 1 provides a brief description of the case study mine (CSM) where the proposed methodology was tested, Sect. 2 uses the results from bonded block pillar strength estimation techniques to evaluate the effect of discontinuities on pillar strength estimation and define pillar average and standard deviation values, Sect. 3 summarizes stochastic finite volume modeling results for pillar stress estimation, and Sect. 4 estimates the CSM pillar probability of failure by combining values obtained in Sects. 2 and 3 using the reliability method. Section 5 presents the results obtained from the LiDAR and photogrammetric surveys and compares those with a fractured pillar stability assessment using the bonded block and filtered discrete fracture network (DFN) model. Finally, a discussion section highlights these results in the context of current ground control management best practices. Results from the proposed design framework provide additional decision-making tools to prevent pillar failure from the design stages by reducing uncertainty. The proposed method enables the integration of pillar design into the risk analysis framework of the mining operation, ultimately improving safety by preventing future pillar collapses.

## Case Study Mine (CSM) and Pillar Strength

The CSM has been described in previous work by the authors [31]. In summary, the operation consists of an underground limestone mine that extracts an approximately 30-m (~ 100 ft) thick and 30° dipping limestone ore body. The mining method is room-and-pillar with eventual stoping. In general, 12.8-m (42-ft) wide and 30-m (100-ft) high stopes are excavated and supported by squared 24-m wide and 30-m (100 ft) tall pillars. These pillars have an approximate W/H ratio of 0.8. Intact rock properties are summarized in Table 1. The orebody rock has an average uniaxial compressive strength of 159.20 MPa with a standard deviation of 21.25 MPa. Four main discontinuity sets were identified in the study area by using conventional mapping and virtual discontinuity mapping from LiDAR scans. The bedding plane set, marked as set 4, reported an approximate trend of N34°E, and a dip of 29° towards SE. The second discontinuity set, referred as set 1, corresponded to a sub-vertical joint set perpendicular to the bedding plane orientation. Sets 2 and 3 were identified as steeply dipping oblique joints. Table 2 summarizes the statistical parameters for the probability distributions for the orientation, trace length, and fractured density measured as number of discontinuities per unit length (P_10_).

**Table 1:** Intact rock property test results from CSM (Monsalve J., Baggett, Bishop, & Ripepi, 2018)

Lithology	Density (ton/m3)		UCS (MPa) *N* = 18		Brazilian tensile strength (MPa) *N* = 18		Young’s modulus (GPa)		Poisson’s ratio	
	**Mean**	**SD**	**Mean**	**SD**	**Mean**	**SD**	**Mean**	**SD**	**Mean**	**SD**
Hangingwall	2.69	0.01	163.74	37.84	11.96	3.14	61.02	6.79	0.19	0.02
Ore Body	2.69	0.01	159.20	21.25	6.30	1.99	64.11	2.37	0.22	0.05
Footwall	2.72	0.01	217.29	36.12	13.72	2.62	61.43	3.15	0.21	0.03

**Table 2:** Statistical Summary of the joint properties for each joint set from CSM (Monsalve J., Baggett, Bishop, & Ripepi, 2018)

Set		S1 *N* = 157	S2 *N* = 127	S3 *N* = 97	S4 (bedding) *N* = 45
Parameters					
Orientation	Dip [°]	88	68	75	29
	Dip Direction [°]	255	348	21	144
	K (Fisher)	103.9	102.4	69.5	197.3
Size	Distribution	Log-normal	Log-normal	Log-normal	Log-normal
	Mean	0.353	0.318	0.018	0.778
	Standard deviation	0.659	0.772	0.749	0.934
Density	Number of fractures per unit length of scan line (P10)	Normal µ = 1.011 σ = 0.495	Min = 0.18 Q2 = 0.576 Median = 1.180 Q3 = 1.577 Max = 1.883	Normal µ = 0.928 σ = 0.492	Normal µ = 0.941 σ = 0.477

Rock properties, discontinuity properties, and mine design characteristics of the CSM were used to estimate pillar strength based on industry standard NIOSH Pillar strength formula. In 2011, the National Institute for Occupational Health and Safety of the United States (NIOSH) published a series of guidelines to help underground stone mining operators to improve their designs and safety of these operations [11]. This document was the product of over 10 years of research on this matter. An empirical equation for pillar strength estimation that considers the impact of discontinuities with respect to the width-to-height ratio and the frequency of such fractures came as a result of this research. The NIOSH equation is mainly applicable to flat-lying, bedded formations with good to very good rock mass quality, room-and-pillar layouts, and mining depths and stresses consistent with those observed in the Eastern and Midwestern United States. This equation is introduced in Eq. 1. This formula contains power constants derived from particular cases from the United States underground stone industry and considers a large discontinuity factor (LDF). This LDF is presented in Eq. 2 and is calculated from a discontinuity dip factor (DDF) and a frequency factor (FF). These two factors were derived from multiple numerical simulations evaluating explicitly the effect of discontinuities on the strength of rock pillars [10, 12].

1 $${S}_{p}=0.65\times UCS\times LDF\times \frac{{W}^{0.3}}{{H}^{0.59}}$$

2 $$LDF=1-DDF\times FF$$

**Fig. 2:**
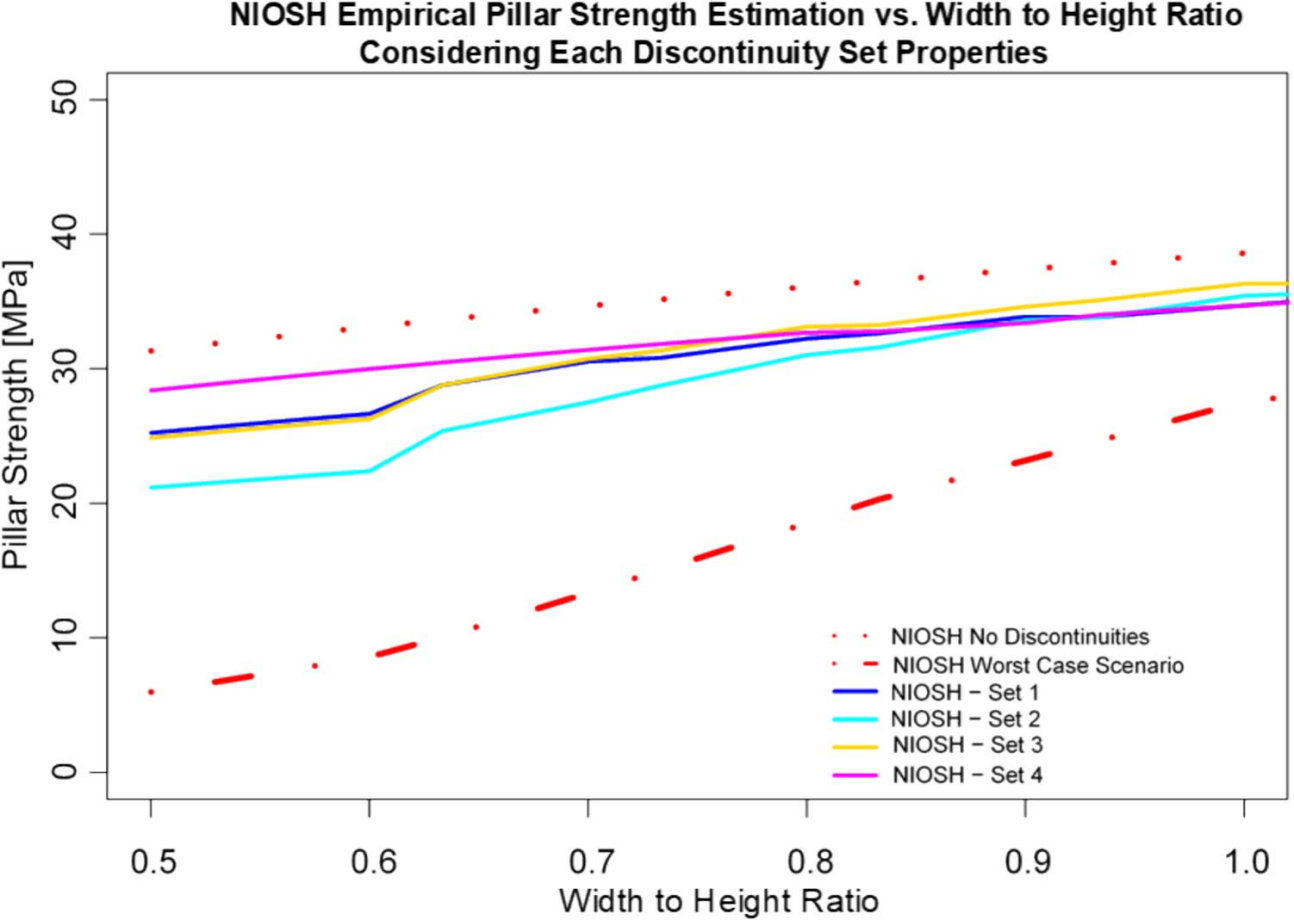
Case study mine pillar strength estimation using the NIOSH empirical formula considering CSM discontinuity set properties

The NIOSH Pillar Strength formula is the only empirical equation that allows to account for the effect of discontinuities in pillar strength. However, it only allows to account for the effect of one discontinuity set at a time. Table 3 summarizes the discontinuity dip factor (DDF) and the frequency factor (FF) for each of the discontinuity sets mapped in the CSM. These values were used to estimate pillar strengths for each discontinuity set, as shown in Fig. 2. This figure also shows the maximum and minimum pillar strength (worst-case scenario) values that can be estimated with this empirical approach. Even though frequency factor and discontinuity dip factor from sets 1, 2, and 3 have the greatest effect on pillar strength at low width-to-height ratios, the LDF obtained from the bedding plane (set 4) was selected as the most representative value for the CSM. This selection was done based on the criteria that the bedding plane is the largest and most continuous fracture set in the rock mass. In addition, structural instability in the CSM has been mostly associated with the bedding plane.

**Table 3:** Discontinuity dip factor and frequency factor for each discontinuity set obtained from mapped discontinuities in the CSM

Set	Discontinuity dip	Discontinuity dip factor	Frequency factor
1	90	0.18	0.63
2	70	0.39	0.39
3	80	0.25	0.39
4	30	0.15	0.63

The NIOSH pillar strength formula was used to estimate a range of pillar strength values for the case study mine. Figure 2 shows a range of possible pillar strength values considering each discontinuity set in the CSM. This figure also shows the maximum and minimum values that can be estimated using this equation. For a pillar with a W/H ratio of 0.8, the expected values lie between 18.7 and 36.1 MPa. Considering that the bedding plane (set 4) is the discontinuity set that mainly controls the stability at the CSM, a pillar strength value around 32.7 MPa can be estimated for this case. These values will be used as a reference in the following sections to evaluate the performance of fractured pillar strength numerical models.

## Stochastic Discrete Element Modeling for Pillar Strength Estimation

### Modeling Methodology

For this section, the bonded block modeling method and the DFN-DEM approach were used to estimate the strength of the pillars using the software 3DEC [22]. The bonded block method (BBM) is a derivation of the discrete element method in which the body of interest is discretized into small polyhedral or tetrahedral elements. Each of the elements composing the geometry are independent blocks bonded to their neighbors by contacts. When the shear or tensile strength of the contacts is surpassed by the stresses, the contact is broken enabling relative displacement between the neighbor blocks. This approach mimics the fracture growth, propagation, and coalescence process that occurs in intact rock material and has been used by multiple authors to simulate laboratory-scale intact rock failure, hard rock pillar failure, and rib and ground support failure and interaction in coal mines [17, 40, 41, 44].

A bonded block model was built to represent intact squared pillars with width-to-height ratios of 0.5, 0.8, and 1.0. Pillar width and height values are shown in Table 4. The pillar was divided into tetrahedral blocks with an edge length of 0.08 times the width of the pillar. This bonded block edge length was determined by performing a trade-off study that determined that this value reduces processing time while providing reasonable strength and failure results [28]. The blocks were zoned using a zone length of 0.35 times the block edge length. The intact blocks were modeled as an elastic material with a Young’s modulus of 64 GPa and a Poisson’s ratio of 0.22, following intact rock properties from Table 1.

**Table 4:** Calibrated parameters for intact pillar bonded block model

Parameter	Model geometry		
Width-to-height ratio	0.5	0.8	1
Pillar width	20	24	25
Pillar height	40	30	25
Bonded block edge length	1.6	1.9	2.0
Zones edge length	0.575	0.672	0.724
	**Intact blocks**		
Constitutive model	Elastic	Elastic	Elastic
Density [ton/m^3^]	2.7	2.7	2.7
Young’s modulus [GPa]	64	64	64
Poisson’s ratio	0.22	0.22	0.22
	**Bonded block contacts**		
BB joint normal stiffness [GPa/m]	500	500	500
BB joint shear stiffness [GPa/m]	80	80	80
BB Phi [°]	25	25	25
BB cohesion [MPa]	15.5	15.3	16.2
Tensile strength [MPa]	17	17	17
	**Discontinuities**		
Joint normal stiffness [GPa/m]	300	300	300
Joint shear stiffness [GPa/m]	30	30	30
Phi [°]	30	30	30
Cohesion [MPa]	0	0	0
	**Strength results**		
Expected NIOSH intact pillar strength	31.34	36.09	38.59
Resulting bonded block intact pillar strength	31.23	36.11	38.49

For each W/H ratio, bonded block contact properties (friction, cohesion, and normal and shear stiffness) were adjusted until pillar peak strength reached a value close to the NIOSH intact pillar strength value when failed in compression. This was done by fixing the top and bottom faces of the pillar in the x and y direction and applying a velocity boundary normal to these faces with a magnitude of 0.1 m/s. Lateral faces of the pillar were unrestrained, allowing displacements on the x, y, and z axis. The stress-deformation curve was extracted for each set of properties and compressive strength and deformation modulus were calculated for each trial. The properties were modified until the strength-deformation curve matched the target strength and deformation modulus. Table 4 summarizes the calibrated bonded block contact properties for each W/H scenario and resulting bonded block intact pillar strength obtained with the calibrated parameters.

**Fig. 3:**
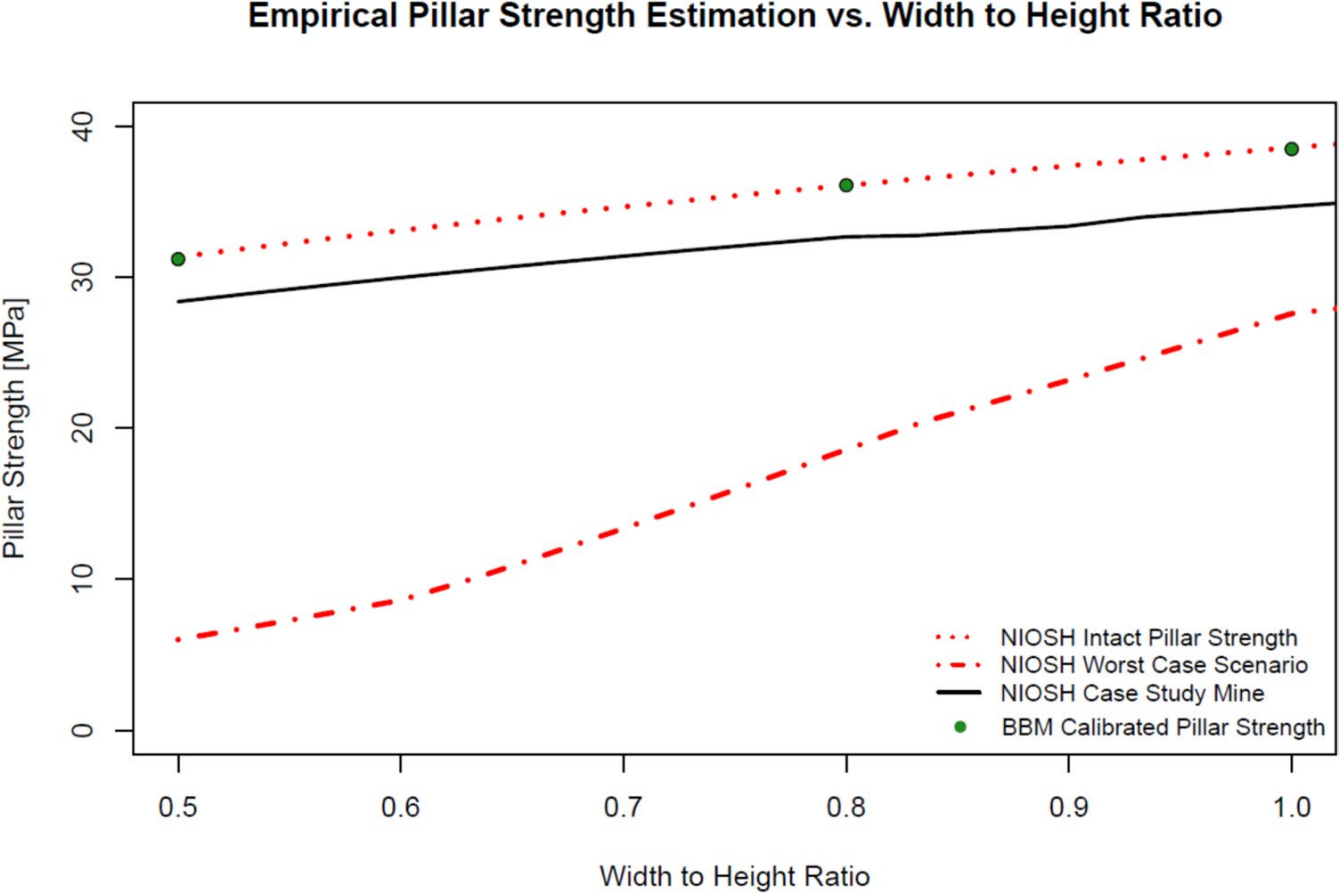
NIOSH intact pillar strength calibration

Figure 3 shows the pillar strength results after calibration for each width-to-height ratio scenario, on top of the pillar strength curve for pillars without discontinuities using the NIOSH empirical equation. In addition, the worst-case scenario and the CSM empirical strength estimation curves are displayed for reference. For the 0.5 W/H ratio pillar, the calibrated model yielded a pillar with an intact pillar strength of 31.23 MPa, producing a 0.35% error in comparison to the NIOSH intact pillar strength estimation. For the 0.8 W/H ratio pillar, the bonded block contact calibration yielded an intact pillar strength of 36.11 MPa, producing a 0.05% error in comparison to the NIOSH intact pillar strength estimation. Finally, for the 1.0 W/H ratio pillar, the bonded block contact calibration yielded an intact pillar strength of 38.49 MPa, producing a 0.25% error in comparison to the NIOSH intact pillar strength estimation.

**Fig. 4:**
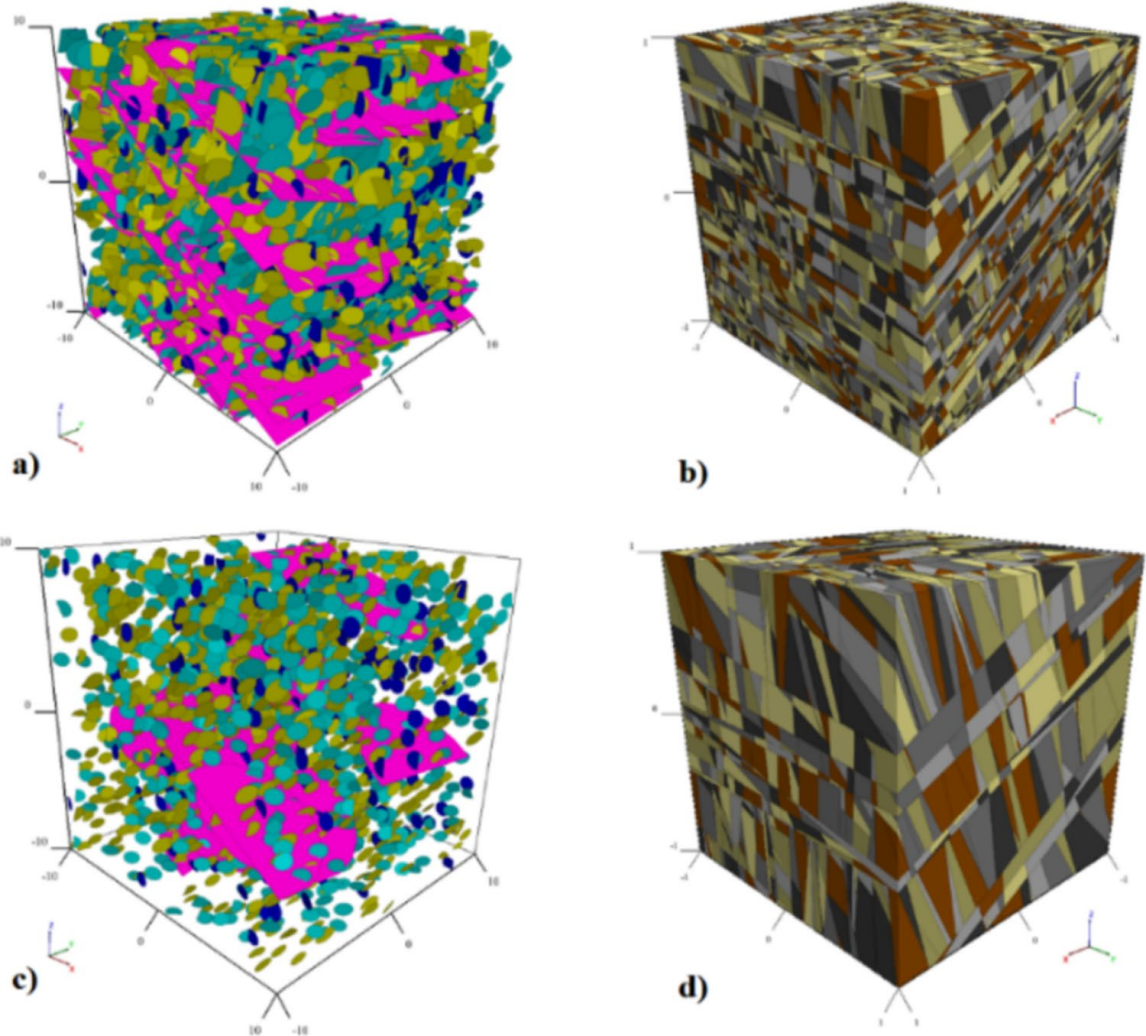
**a** Dense DFN model, **b** fractured rock mass model generated from the dense DFN, **c** filtered DFN model, and **d** fractured rock mass model generated from the filtered DFN

DFN models were created in the DEM software 3DEC using the properties described on Table 2. A detailed description of the process for the DFN generation is provided in Monsalve et al. [30]. Two DFN models were defined, a dense DFN model that accounted for all mapped discontinuities (Fig. 4a) and a filtered DFN model which only considered those larger than 2 m (12.5% the pillar width) (Fig. 4b). The filtered DFN was used to evaluate the effect of discontinuities on pillar strength. Discontinuities smaller than 2 m were filtered out of the model to optimize processing times while still capturing structural impact on the rock mass. This approach follows the recommendation from [47] that acknowledges that including every discontinuity in a numerical model is computationally expensive and time-consuming. Therefore, a simplified approach that effectively represents fractured rock mass behavior at the problem’s scale is necessary.

**Fig. 5:**
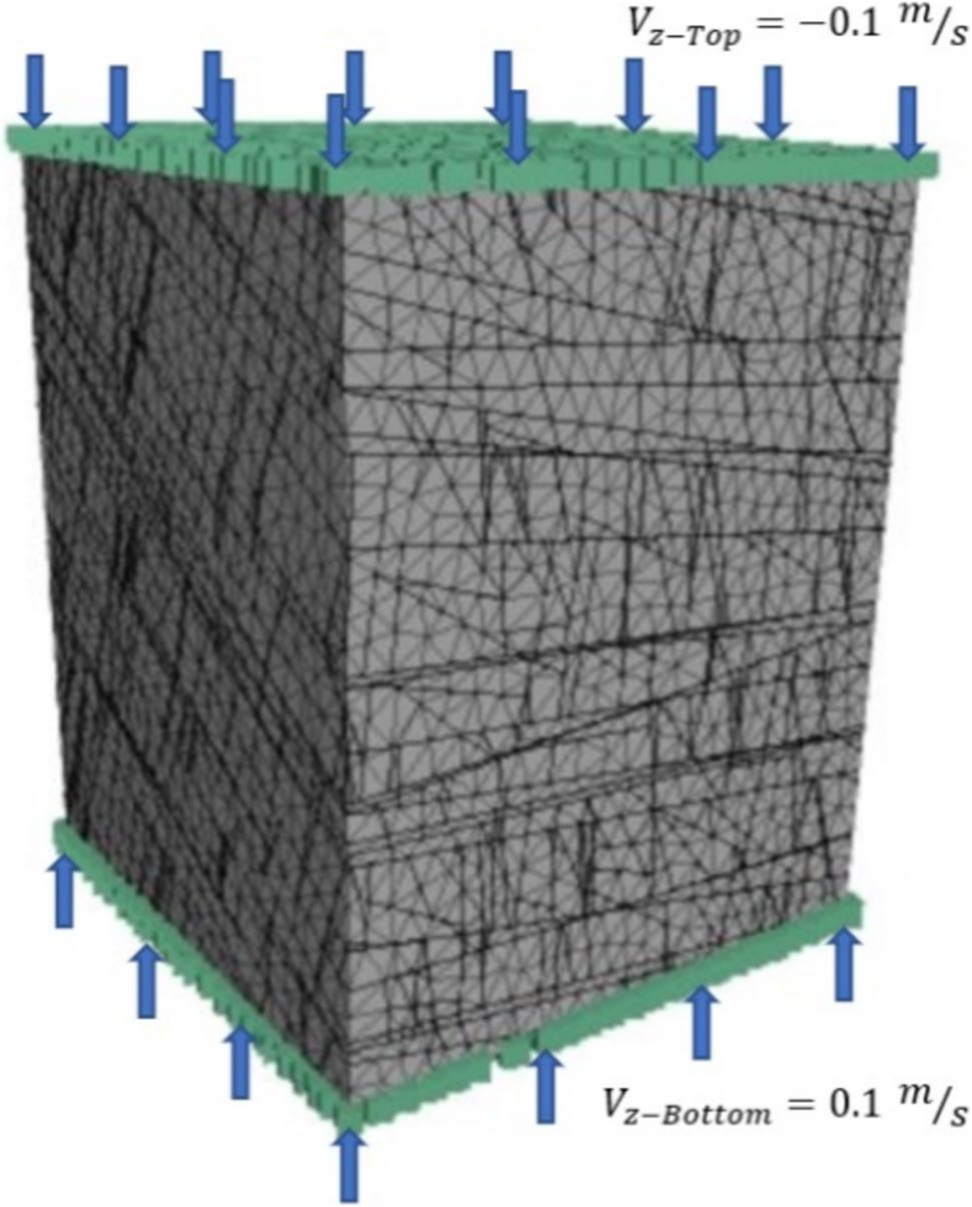
Pillar model setting and boundary conditions

**Fig. 6:**
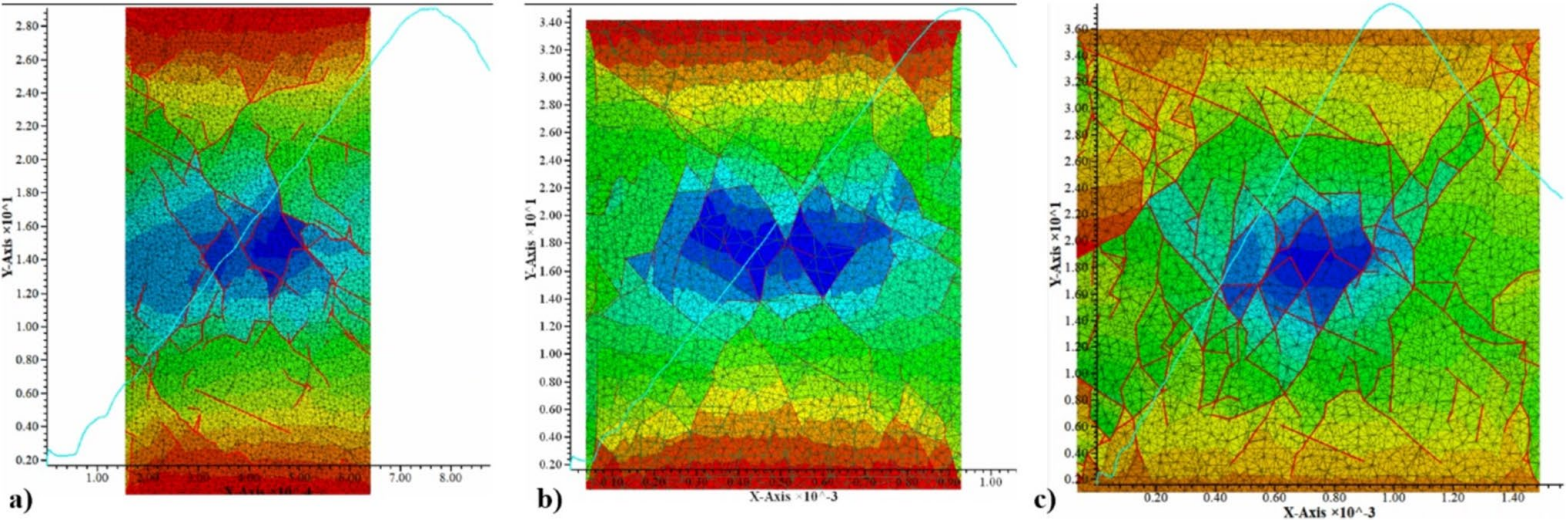
Stochastic pillar strength estimation using the bonded block method and a filtered DFN. **a** Pillar strength test a 0.5 width-to-height ratio pillar, **b** pillar strength test a 0.8 width-to-height ratio pillar, **c** pillar strength test a 1.0 width-to-height ratio pillar

Once intact pillar bonded block contact properties were calibrated to the NIOSH empirical equation values, the effect of discontinuities on pillar strength was evaluated. This process was done by intercepting the intact pillar model with the filtered DFN model and simulating the failure of the pillar in compression as described in Fig. 5. A total of 15 stochastic realizations were run for each of the evaluated pillar W/H ratios (0.5, 0.8, and 1.0). The stress-displacement curves were recorded for each simulation, as well as the processing time per simulation. Figure 6 shows the stress deformation curve for a different realization of each of the W/H ratios. The red lines indicate pre-existing fractures and bonded block contact breakage due to shear or tensile failure on a cross section of the tested pillars.

Results from the simulations were recorded, and fractured pillar strength and deformation modulus were calculated from the stress-displacement curves. A fracture pillar strength summary was imported into RStudio [38] and results were compared with the NIOSH pillar strength assuming no discontinuities and the NIOSH CSM pillar strength. The RStudio “fitdistrplus” library was used to fit probability distribution functions for each fractured pillar strength by W/H ratio, and mean, standard deviation, and coefficient of variation (COV) values were calculated for each case.

### Pillar Strength Estimation Results

**Fig. 7:**
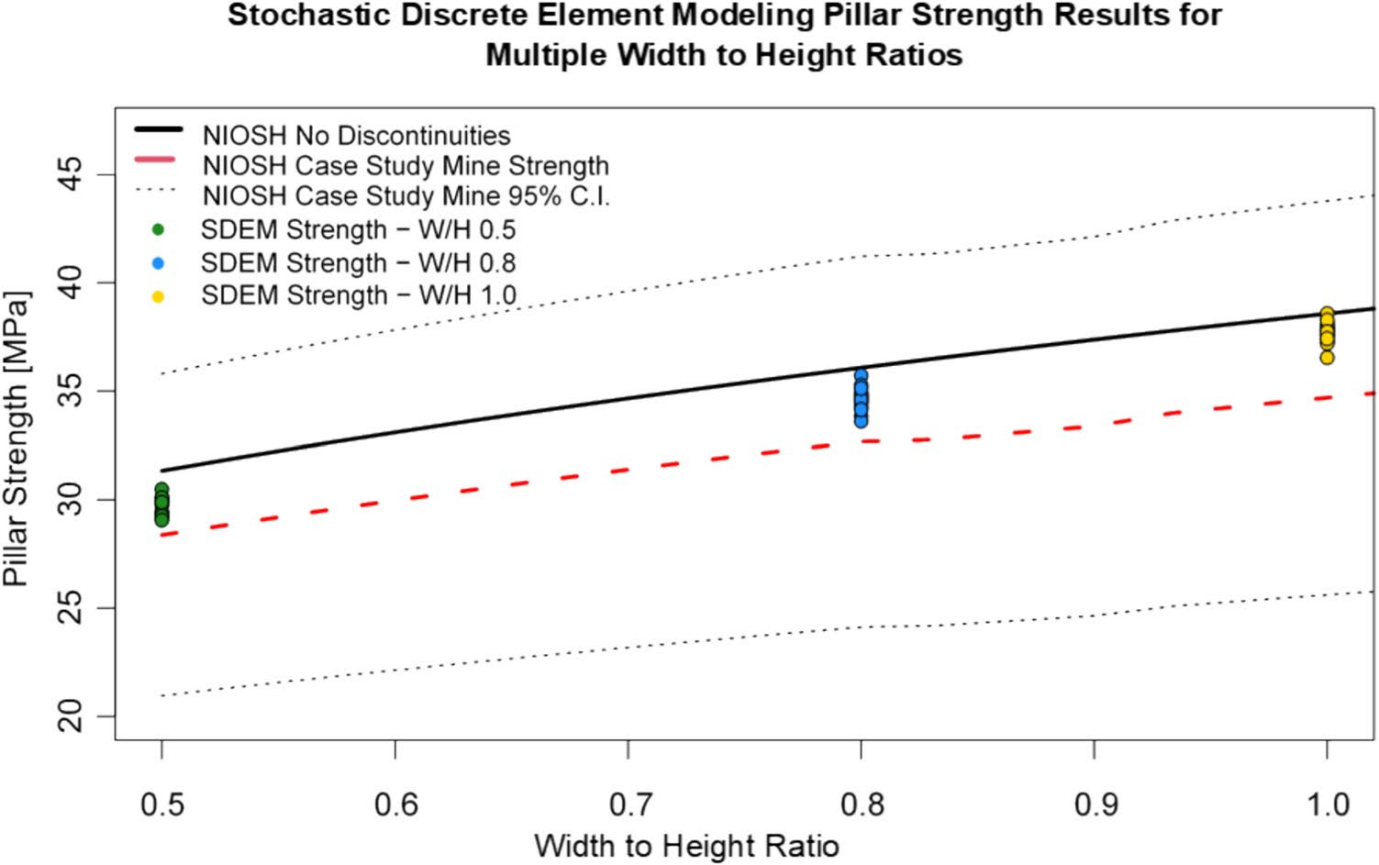
Stochastic discrete element modeling pillar strength results for multiple width to height ratios compared with NIOSH empirical pillar strength estimations

Figure 7 summarizes the results from the stochastic discrete element modeling analysis and compares numerical modeling results with the NIOSH empirical pillar strength estimations. The black dotted line represents the 95% confidence interval (C.I.) for the NIOSH CSM strength curve by considering intact rock uniaxial compressive strength variability. Results indicate that the presence of discontinuities does affect pillar strength for all W/H ratios. In all three cases, the average fractured pillar strength yielded values that were lower than the intact pillar strength calibrated value as indicated in Table 5. For the 0.5 W/H ratio pillar, the fracture pillar strength was 95% the value of the NIOSH intact pillar strength; for the 0.8 W/H ratio pillar, the fracture pillar strength was 96% the strength of the intact pillar; and for the 1.0 W/H ratio pillar, the strength of the pillar considering the effect of discontinuities was 98% the strength of the intact pillar. Discontinuities had less effect on the pillars with higher W/H ratios.

**Table 5:** Stochastic DEM fractured pillar strength comparison with NIOSH empirical strength results

W/H ratio	Fractured pillar strength			Fractured pillar strength/NIOSH intact pillar strength	Fractured pillar strength/NIOSH CSM pillar strength
	Average [MPa]	Standard deviation [MPa]	COV [%]		
0.5	29.7	0.42	1.41%	0.95	1.05
0.8	34.6	0.54	1.56%	0.96	1.06
1	37.7	0.49	1.29%	0.98	1.08

Results were also compared with the NIOSH CSM pillar strength values. It was observed that for all three W/H ratio cases, the stochastic DEM fractured pillar strength was greater than the values estimated using the NIOSH empirical strength formula. The stochastic discrete element model pillar strengths were 5%, 6%, and 8% higher than the NIOSH CSM pillar strength for the 0.5, 0.8, and 1.0 W/H ratio pillars, respectively. All stochastic DEM pillar strength estimations were within the 95% C.I. of the NIOSH CSM pillar strength estimation.

Numerical models used to derive the NIOSH LDF assumed that discontinuities were continuous across the entire pillar producing conservative results [13]. The DFNs used in these simulations considered the effect of joint size in pillar behavior. This consideration may enable the representation of rock bridges within the rock pillar, which may yield higher strengths than assuming fully continuous fractures. The effect of discontinuity size on pillar strength effect has been documented in the past by other authors [49].

**Fig. 8:**
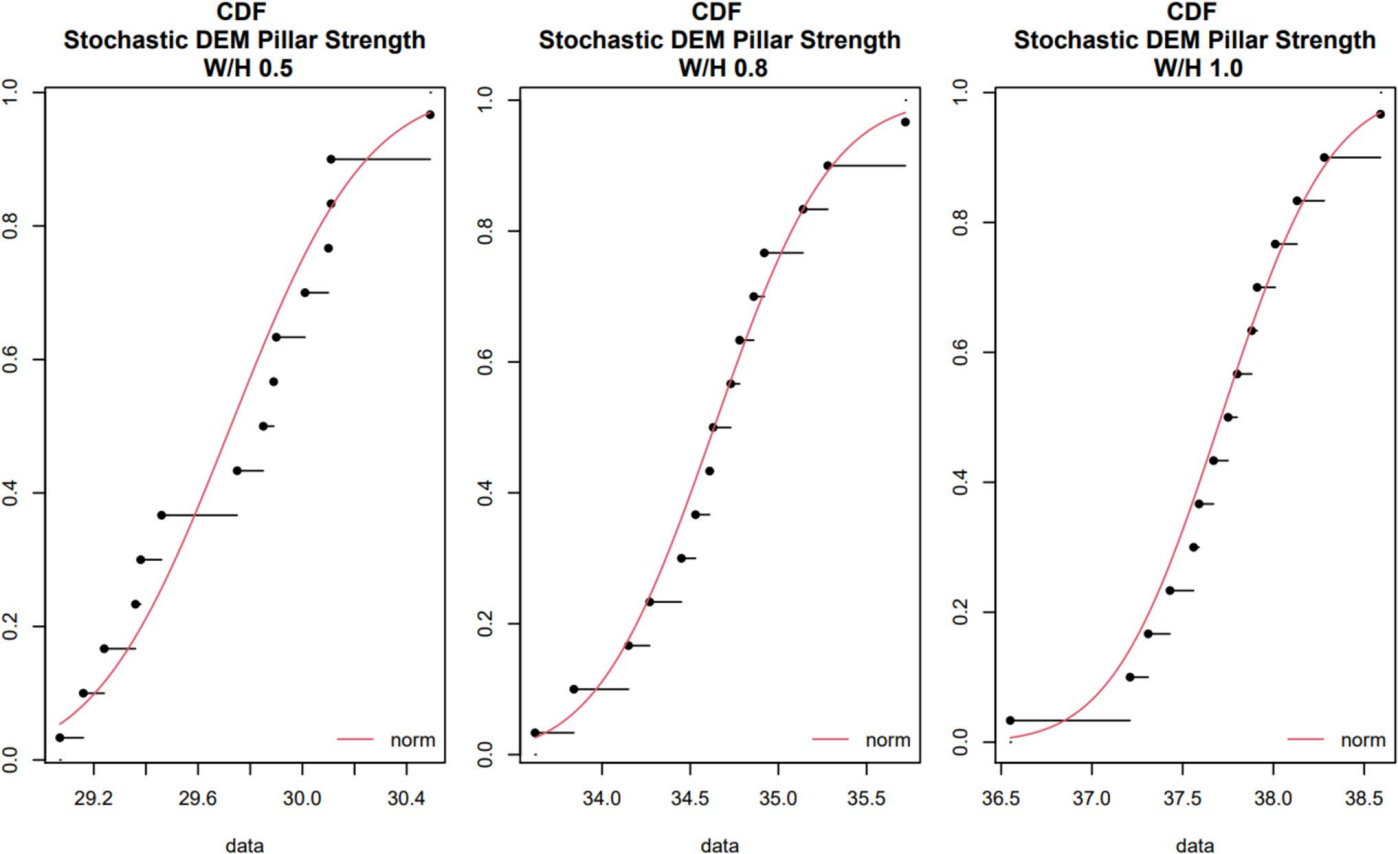
Theoretical and experimental cumulative density functions for fractured pillar strengths for different W/H ratios

The stochastic analysis enabled to characterize the effect of discontinuities’ variability on pillar strength variability. Figure 8 compares the experimental cumulative density functions for all fractured pillar strengths for each W/H ratio. The red line represents the normal theoretical cumulative density function (CDF) for each case. All stochastic DEM pillar strength empirical CDFs show good agreement with the normal CDF model, concluding that the stochastic DEM pillar strength values were all normally distributed. Table 5 shows the average and standard deviation fractured pillar strength for each W/H ratio. The COV for the stochastic DEM pillar strength estimations were 1.41% for the 0.5 W/H ratio pillar, 1.56% for the 0.8 W/H ratio pillar, and 1.29% for the 1.0 W/H ratio pillar.

**Fig. 9:**
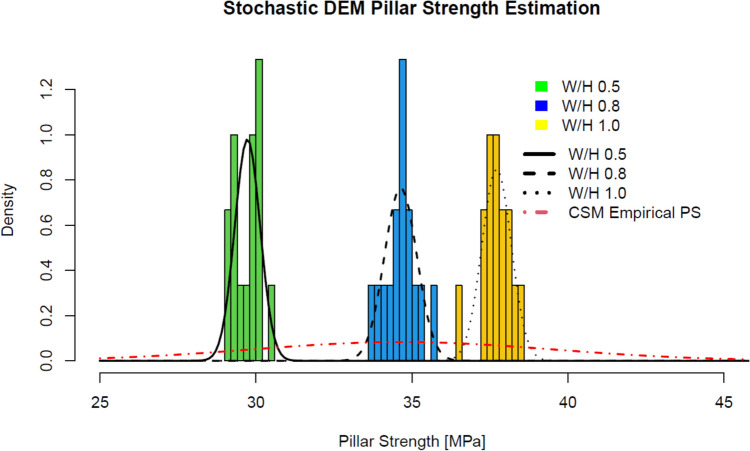
Histograms and probability distribution functions for each W/H ratio using the stochastic DEM pillar strength estimation approached compared to the NIOSH empirical pillar strength distribution

Figure 9 shows the histograms and the fitted probability distribution functions (PDFs) for the stochastic DEM pillar strength estimation for each pillar W/H ratio. It is possible to observe that all fitted normal distributions align well with the SDEM pillar strength histograms. These results are overlaid with the PDF of the NIOSH CSM pillar strength considering intact rock variability. As shown in Table 1, intact rock UCS has a standard deviation of 21.25 MPa. By considering this value on the NIOSH empirical pillar strength equation, the NIOSH CSM pillar strength standard deviation yields a value of 4.36 MPa, as recorded in Table 5. Similarly, the NIOSH CSM pillar strength COV yields a value of 13.3%.

A total computing time of 293 h and 55 min was recorded for all 45 simulations, averaging 6 h and 26 min per each simulation. Table 6 summarizes total and average computing time for each pillar W/H ratio. As previously mentioned, a total of 15 realizations were run for each scenario. The 0.5 W/H ratio pillars took on average 8 h and 59 min per model, the 0.8 W/H ratio pillar simulations took on average 5 h and 4 min, and the 1.0 W/H ratio pillars took 5 h and 4 min per simulation. Slender pillars took longer processing times since the bonded block size, and zone size were a function of the pillar width as shown in Table 6. Therefore, slender pillars had more elements for computation. It is worth mentioning that this total computing time does not consider all the time expended in model calibration efforts.

**Table 6:** Computing time results for the stochastic DEM pillar strength estimation approach for 15 simulations for each W/H ratio

Computing time	Width to height ratio			Total
	**0.5**	**0.80**	**1.00**	
**Total**	143 h 58 min	78 h 56 min	71 h 0 min	293 h 55 min
**Average**	8 h 59 min	5 h 15 min	5 h 4 min	6 h 26 min

In the context of this work, results from the stochastic DEM for pillar strength determination are valuable since they allowed it to estimate a more realistic pillar strength range of values for different pillar geometries considering the CSM site-specific conditions. Results from this section will be used to estimate pillar probability of failure in the following sections. For the probability estimation section, the best estimate for pillar strength will be the average stochastic DEM pillar strength estimated value (34.6 MPa for 0.8 W/H ratio pillars). For pillar strength standard deviation, the selected value will be the NIOSH CSM pillar strength standard deviation (4.36 MPa).

## Stochastic Finite Volume Modeling for Pillar Stress Estimation

### Modeling Methodology

**Fig. 10:**
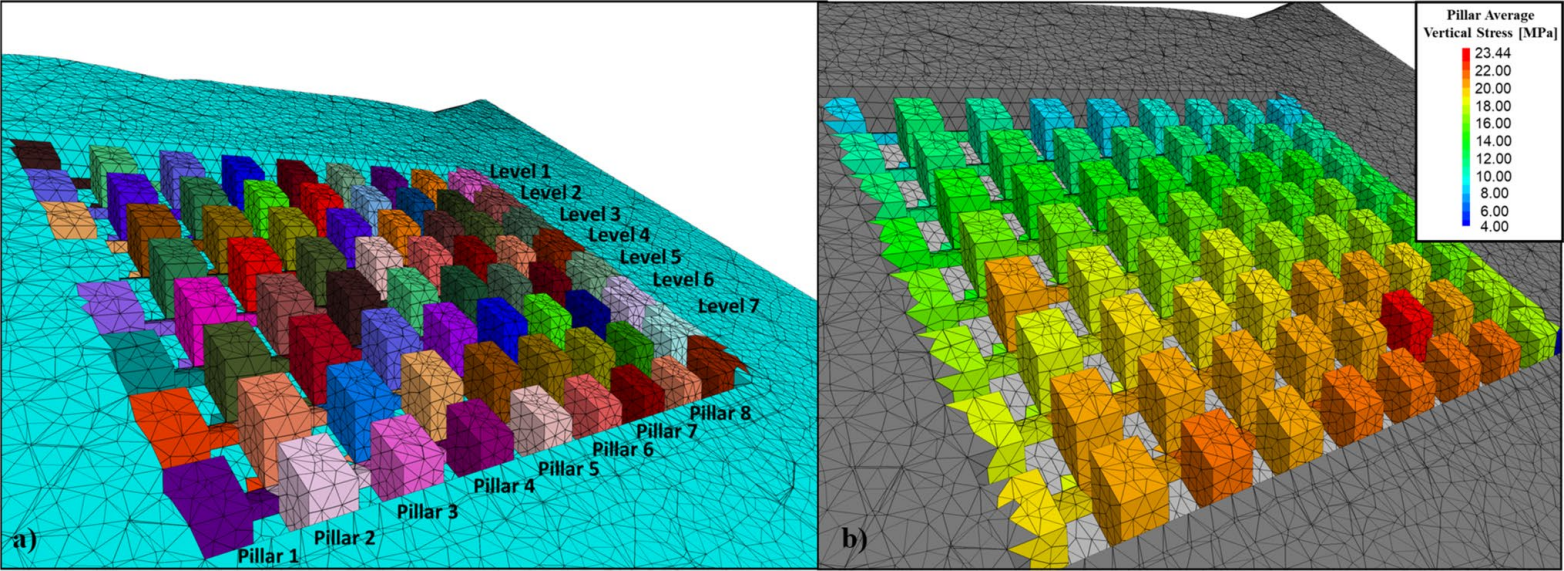
Finite volume numerical analysis to estimate stresses around the CSM

A 3D finite volume pillar stress model for a simplified section of the CSM was performed using the software 3DEC. This model used an elastic modeling approach considering rock mass properties reported in previous work by [31]. For the simulation, three stress scenarios were defined due to the lack of information with regard to in situ stress measurements. Table 7 summarizes each of the three scenarios. The point estimate method was used to estimate the effect of rock mass elastic properties variability on pillar stress distribution. A total of 24 simulations were run, 8 per stress scenario. Results from each of the eight simulations were used to estimate average and standard deviation vertical stress values for each pillar in the simplified model. Figure 10a shows each of the pillar marked on the simplified mine model. Pillars are labeled from 1 to 72, starting at the bottom left of the pillar arrangement. Figure 10b presents the average vertical pillar stress for one of the stress scenarios.

**Table 7:** Horizontal to vertical stress ratios for the different stress scenarios

Scenario 1	${\sigma }_{H}={\sigma }_{v}$	${\sigma }_{h}={\sigma }_{v}$
Scenario 2	${\sigma }_{H}={\sigma }_{v}$	${\sigma }_{h}={0.5 \sigma }_{v}$
Scenario 3	${\sigma }_{H}={1.5 \sigma }_{v}$	${\sigma }_{h}={1.5 \sigma }_{v}$

### Stress Estimation Results

Results from this work enabled to estimate average and standard deviation vertical stress for each individual pillar within the simplified mine model under evaluated stress scenario. It was observed that vertical pillar stress increased with depth due to the dipping nature of the deposit. The effect of surface topography was also evidenced in these simulations since pillar stress magnitude followed the surface elevation contour trends. Pillars on the abutments of the pillar arrangement presented lower stresses, possibly due to the pressure arch load developed due to the self-supporting capacity of the ground on the abutments. Stress scenario 3 ($${k}_{o}=1.5)$$ yielded the highest values for average pillar stress with a magnitude of 23.44 MPa, followed by stress scenario 1 ($${k}_{o}=1.0)$$ which reported a maximum pillar stress magnitude of 19.49 MPa, and then by stress scenario 2 ($${k}_{o}=0.5)$$ with a maximum pillar stress value of 17.84 MPa. Another important observation was that pillars that presented higher stresses, where the same ones that reported higher standard deviation values. Mean and standard deviation values for average vertical stress for each pillar under each stress scenario will be used to estimate pillar probabilities of failure in the following section.

## Pillar Probability of Failure Estimation

### Modeling Methodology

The reliability analysis method is a risk analysis approach that allows to estimate the probability of failure of certain system by considering the probability distribution functions of the different variables affecting the system. This section will describe the calculation procedure used to estimate pillar probability of failure in the CSM based on the results obtained from the previous two sections. Results obtained from these calculations are presented and will be discussed in the context of the mining operation, current pillar design standards, and ground control management best practices.

The simplified pillar stress estimation model was used as the base model to estimate pillar probability of failure. The model geometry and its results, as shown in Fig. 10, were used to (1) obtain pillar stress average and standard deviation for each pillar in the pillar arrangement and (2) report pillar probability of failure results after calculation. In the pillar design context, a factor of safety is usually defined as the relation between pillar strength and the normal stresses applied to it, as shown in Eq. 3.

3 $$F.S.=\frac{Strength}{Stress}=\frac{{S}_{p}}{{\sigma }_{p}}$$

From this approach, a limit state function $g$ can be defined as the difference between pillar strength and pillar stress, as indicated in Eq. 4.

4 $$g\left({S}_{p},{\sigma }_{p}\right)= {S}_{p}-{\sigma }_{p}$$

Considering this limit state function, it is possible to define a failure set when the values for pillar strength and stress yield negative values for the limit state function, as shown in Eq. 5.

5 $$Failure=\left\{g\left({S}_{p},{\sigma }_{p}\right)<0\right\}$$

Similarly, a probability of failure can be defined as the probability for the function $g\left({S}_{p},{\sigma }_{p}\right)$ to take negative values, as defined in Eq. 6.

6 $${P}_{f}=P(g\left({S}_{p},{\sigma }_{p}\right)<0)$$

Given the limit state function, a reliability index $(\beta )$ can be defined as the ratio between the mean value of the limit state function, and its standard deviation, as shown in Eq. 7.

7 $$\beta =\frac{{\mu }_{g}}{{\sigma }_{g}}$$

The mean value of the limit state function can be found by simply evaluating the function in the variables mean values, as:

8 $${\mu }_{g}=g({\mu }_{{S}_{p}},{\mu }_{{\sigma }_{p}})$$

In the pillar design case, the mean value for the limit state function can be evaluated as indicated in Eq. 9.

9 $${\mu }_{g}={\mu }_{{S}_{p}}-{\mu }_{{\sigma }_{p}}$$

The variance of the limit state function can be found by using the first-order second moment, derived from the Taylor’s series expansion of $g\left({S}_{p},{\sigma }_{p}\right)$ about ${\mu }_{{S}_{p}} and {\mu }_{{\sigma }_{p}}$. By using this approximation, the variance of the limit state function can be calculated as shown in Eq. 10.

10 $${{\sigma }_{g}}^{2}={\nabla }_{X}{g}^{T}{{\Sigma }_{XX}\nabla }_{X}g$$

where ${\Sigma }_{XX}$ is the covariance matrix, and ${\nabla }_{X}g$ is a partial derivative vector of $g$. Equations 11 and 12 show how to calculate the ${\Sigma }_{XX}$ and ${\nabla }_{X}g$, respectively.

11 $${\Sigma }_{XX}=\left[\begin{array}{cc}{{\sigma }_{{S}_{p}}}^{2}& {{\rho \sigma }_{{S}_{p}}\sigma }_{{\sigma }_{p}}\\ {{\rho \sigma }_{{S}_{p}}\sigma }_{{\sigma }_{p}}& {{\sigma }_{{\sigma }_{p}}}^{2}\end{array}\right]$$

12 $${\nabla }_{X}g=\left[\begin{array}{c}{~}^{\partial g}\!\left/ \!{~}_{\partial {S}_{p}}\right.\\ {~}^{\partial g}\!\left/ \!{~}_{\partial {\sigma }_{p}}\right.\end{array}\right]$$

For this particular problem, if it is assumed that the pillar strength is uncorrelated with the pillar stress, the expression for the standard deviation for the limit state function can be calculated as expressed in Eq. 13.

13 $${\sigma }_{g}=\sqrt{{{\sigma }_{{S}_{p}}}^{2}+{{\sigma }_{{\sigma }_{p}}}^{2}}$$

Additionally, if the pillar strength and pillar stress are normally distributed, the limit state function is also normally distributed. In that case, the probability of failure can be directly computed as:

14 $${P}_{f}=1-NORMDIST(\beta )$$

The software RStudio [38] was used to compute the probability of failure for each pillar. Results for the stochastic pillar stress estimation model produced a 72-row × 2-column matrix where each row represented each pillar in the pillar arrangement following the labeling indicated in Fig. 10a. The first column of that resulting vector reported the average vertical pillar stress, and the second column contained the standard deviation for each pillar. A total of three matrixes were obtained, one for each pillar stress scenario evaluated, as described in Table 7.

Results from the stochastic DEM pillar strength estimation stage and those of the stochastic FVM stress estimation were used to estimate probability of failure for each individual pillar using Eqs. 5, 7, 11, and 12. A factor of safety was also calculated for each pillar according to Eq. 1. For these calculations, it was assumed that pillar strength and pillar stress were not correlated and that all pillars in the pillar arrangement had the same W/H ratio. Therefore, the average and standard deviation pillar strength was the same for all pillars. The average and standard deviation vertical pillar stress were variable according to the stress simulation stochastic analysis.

### Probability of Failure Estimation Results

**Fig. 11:**
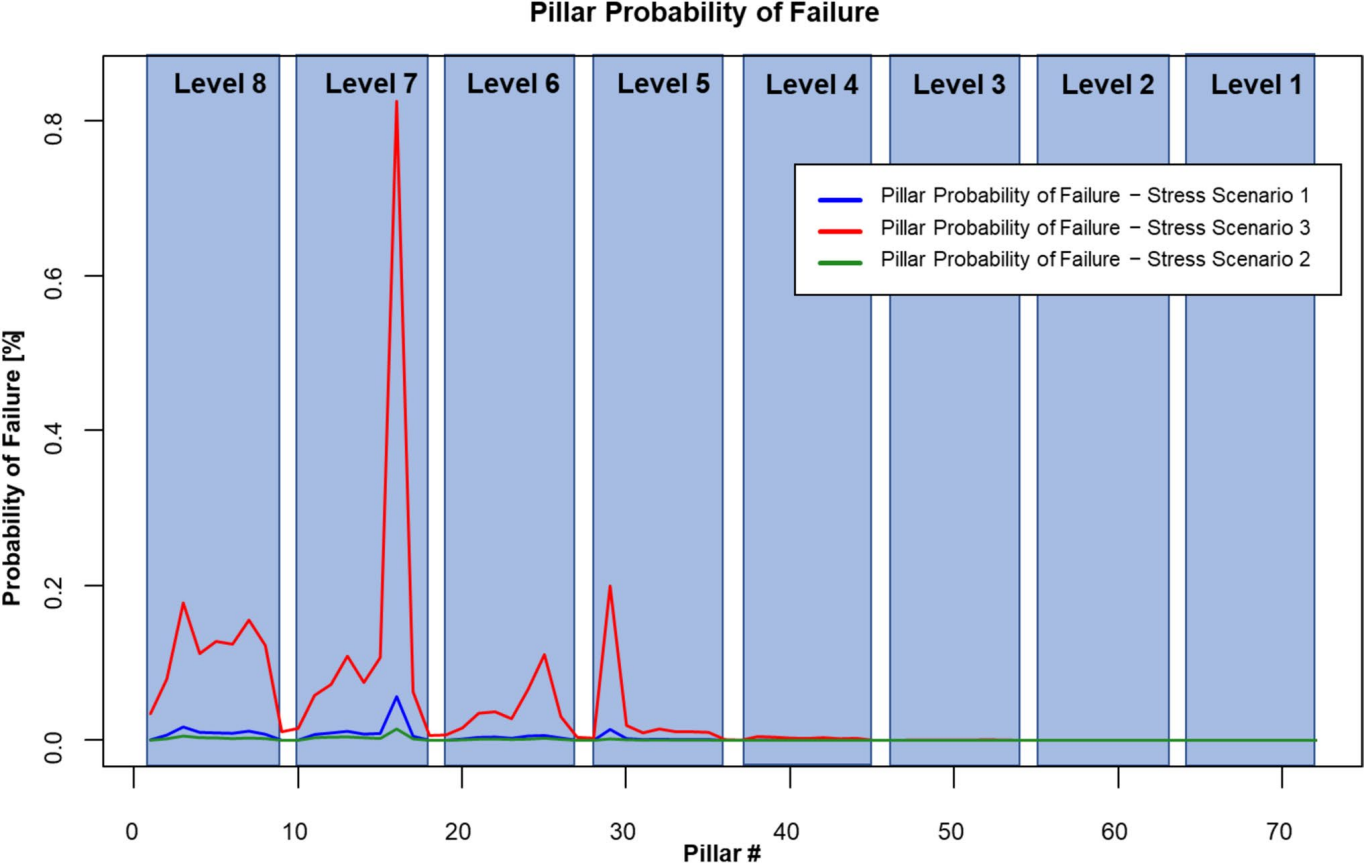
Pillar probability of failure for each stress scenario

A probability of failure and a factor of safety were calculated for each pillar at each stress scenario. Figure 11 summarizes the probability of failure for each pillar in the CSM simplified pillar model. Pillars are labeled from 1 to 72 and are highlighted by level. This means that pillars contained in the same level are at approximately the same depth from the mine portal. The maximum probabilities of failure were 0.056%, 0.014%, and 0.825% for stress scenarios 1, 2, and 3, respectively. In all cases, pillar #16 was the pillar with the highest probability of failure. Stress scenario 2 ($${k}_{o}=0.5)$$ yielded the lower probability of failure values, whereas stress scenario 3 ($${k}_{o}=1.5)$$ yielded the highest probabilities of failure. From levels 1 to 4, all pillars seem to have a negligible probability of failure in all stress scenarios. However, for stress scenario 3, pillar #29 located at level 5 presents a probability of failure of 0.199%, a value significantly higher than those obtained for the other two stress scenarios.

**Fig. 12:**
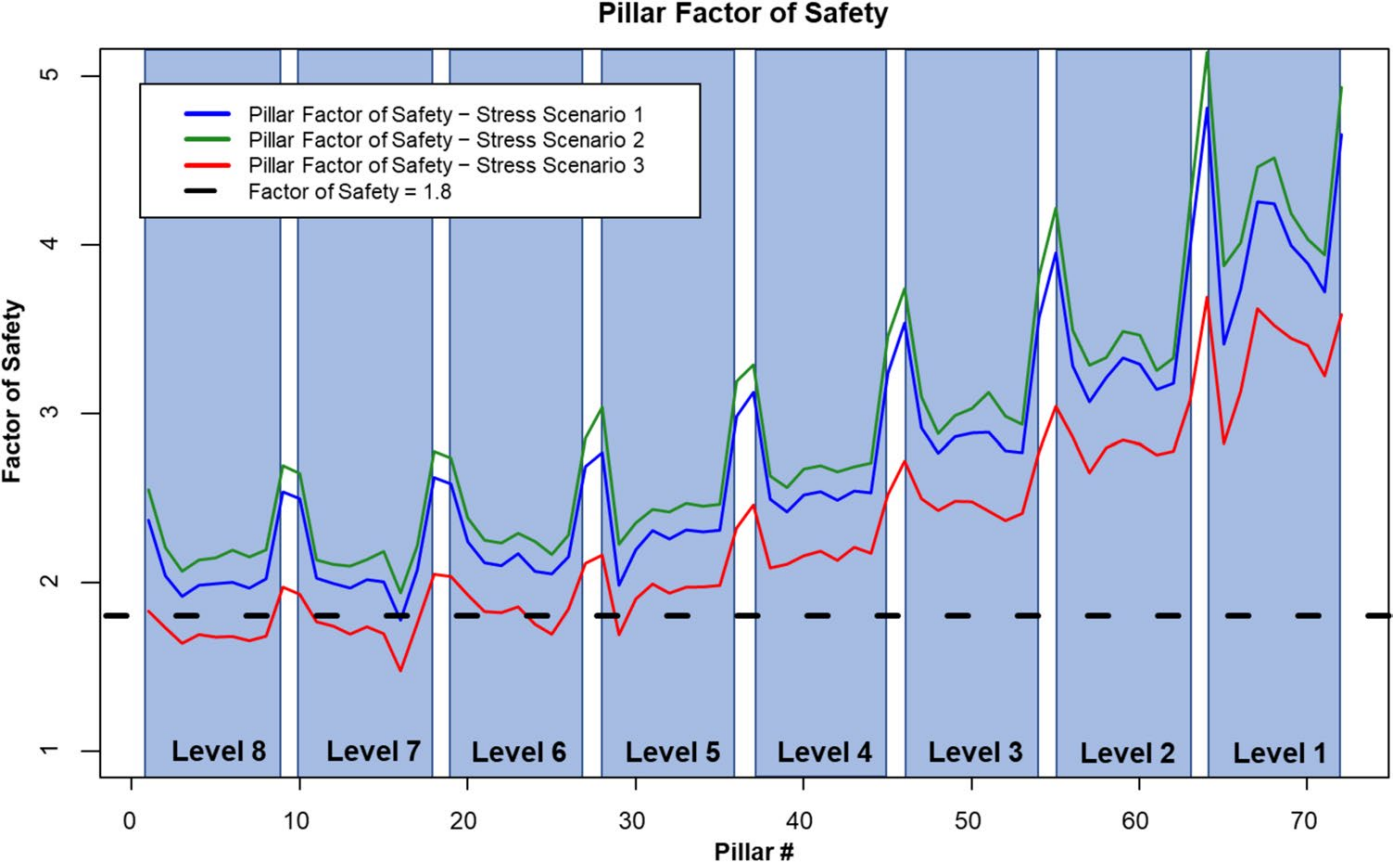
Pillar factor of safety for each stress scenario

Current limestone pillar design guidelines do not define probability of failure as an acceptance criterion [1]. Therefore, there is not an actual definition of what an acceptable probability of failure value could be for underground stone mines. Current guidelines define a 1.8 factor of safety as the acceptable design parameter for pillars in limestone mines in the United States. Figure 12 presents the calculated pillar factor of safety for the CSM for each stress scenario evaluated. For stress scenario 2 (${k}_{o}=0.5$), all pillars presented a factor of safety above the design standard of 1.8. The minimum factor of safety obtained in this case was 1.94, calculated for pillar 16. For stress scenario 1 (${k}_{o}=1.0$), only one pillar did not meet the design criteria of 1.8. The pillar that did not meet this criterion was pillar #16 in the level 7, with a factor of safety of 1.77. In the case of stress scenario 3 (${k}_{o}=1.5$), 17 pillar yielded factor of safety values lower than the minimum acceptable value of 1.8. Table 8 summarizes the factor of safety values and probability of failures for each of the 17 pillars with a factor of safety lower than 1.8 assuming the worst-case scenario for stress conditions. In this case, the pillar with the lowest factor of safety was again pillar #16, with a factor of safety of 1.48.

**Table 8:** Computing time results for the stochastic DEM pillar strength estimation approach for 15 simulations for each W/H ratio

Stress scenario 3—${{\varvec{\sigma}}}_{{\varvec{H}}}={{\varvec{\sigma}}}_{{\varvec{h}}}={1.5\boldsymbol{ }{\varvec{\sigma}}}_{{\varvec{v}}}$		
**Pillar #**	**Factor of safety**	**Probability of failure [%]**
2	1.73	0.08
3	1.64	0.18
4	1.69	0.11
5	1.68	0.13
6	1.68	0.12
7	1.66	0.16
8	1.68	0.12
11	1.77	0.06
12	1.74	0.07
13	1.70	0.11
14	1.74	0.07
15	1.70	0.11
16	1.48	0.83
17	1.76	0.06
24	1.75	0.07
25	1.70	0.11
29	1.69	0.20

**Fig. 13:**
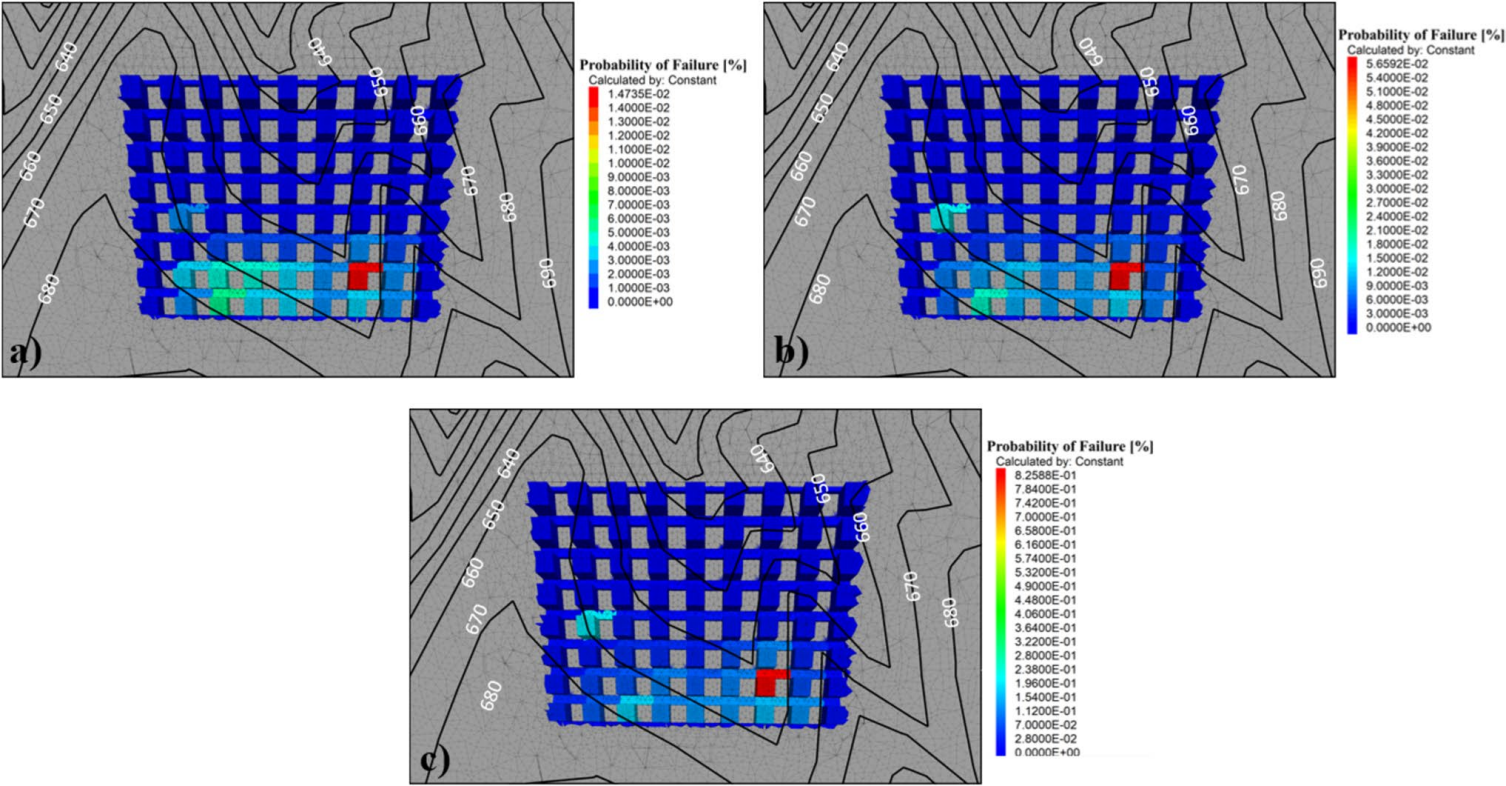
Top view of the simplified CSM model colored by probability of failure for each stress scenario. **a** Stress scenario 2, ko = 0.5; **b** stress scenario 1, ko = 1.0; and **c** stress scenario 3, ko = 1.5

Figure 13 shows a plan view of the CSM pillar arrangement overlaid with the surface contour elevations. Pillars are mapped by their calculated probability of failure for each different stress scenario. Figure 13a represents the results from stress scenario 2 (${k}_{o}=0.5$), Fig. 13b shows the results from stress scenario 1 (${k}_{o}=1.0$), and Fig. 13c shows the results from stress scenario 3 (${k}_{o}=1.5$). Pillars with higher probabilities of failure were the same in all stress scenarios. However, the probability of failure value was in a different scale. Pillars with higher probabilities of failure are those that also presented higher stress magnitudes and variability. These pillars were also associated with the surface change of topography and the dipping nature of the deposit.

**Fig. 14:**
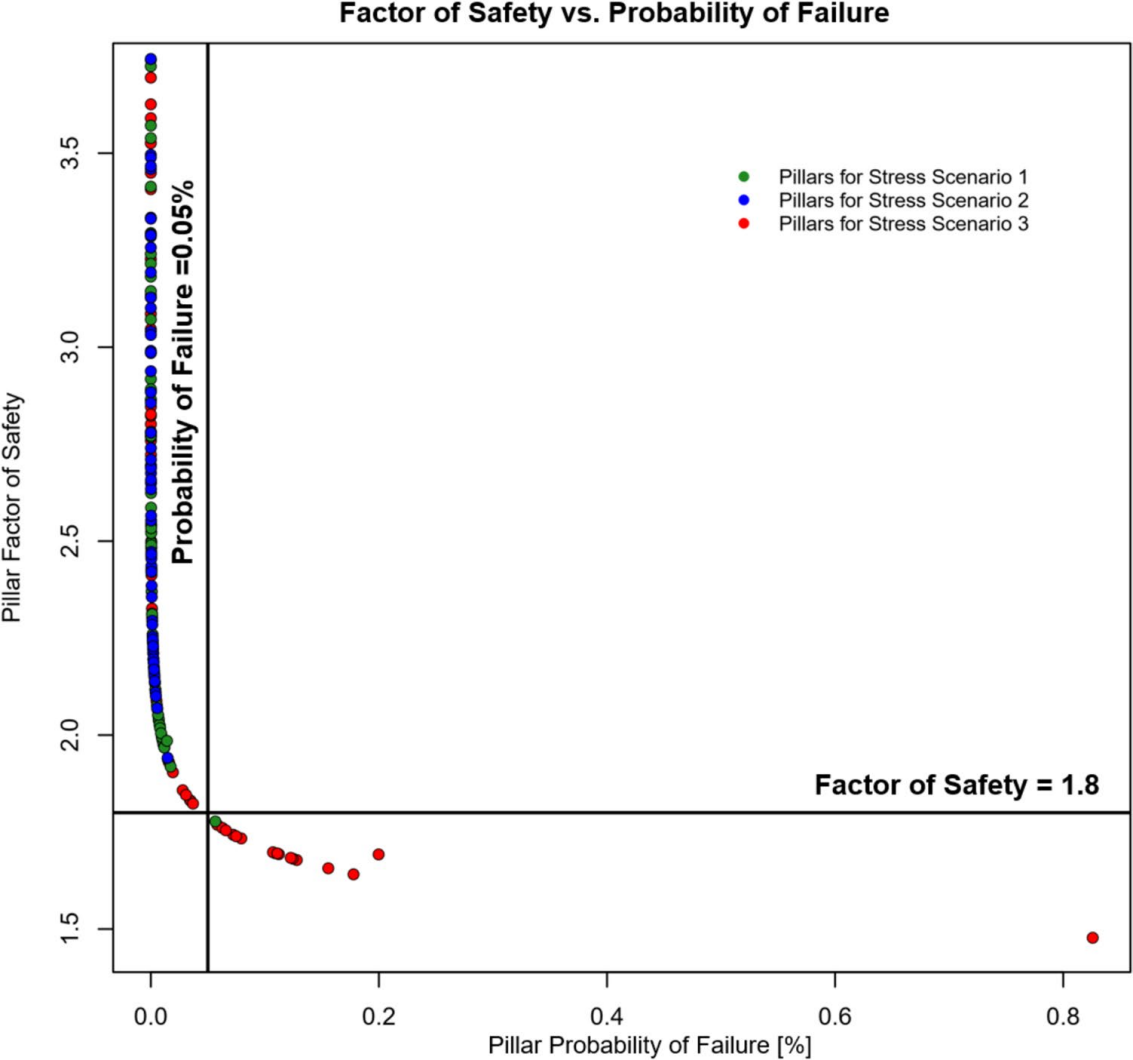
Pillar factor of safety vs. probability of failure

Even though there is not any defined acceptable probability of failure in underground stone mines, the industry accepted guideline of a 1.8 factor of safety, and the design methodology presented in this work can help in determining what an acceptable value for pillar probability of failure could be. Figure 14 presents a plot indicating the factor of safety and the probability of failure for all the analyzed pillars at the different stress conditions. In this case, the factor of safety follows a negative logarithmic trend with respect to the probability of failure. Considering the trend of the plotted data, and the 1.8 factor of safety, a maximum acceptable probability of failure of 0.05% could be defined.

## Pillar Stability Assessment and Model Validation

### Modeling Methodology

The only method to determine the real ultimate pillar strength is by subjecting this element to failure loads. In the past, authors have performed in situ large-scale testing to determine in situ coal strength and deformation properties [2]. However, such analyses are cost-prohibitive and time-consuming, and the resulting data cannot be used in other sites. In the context of large opening underground stone mines, these types of tests are simply untenable due to the challenges and risks associated with the collapse of a large hard rock pillar. Other authors have used the experience obtained from real pillar collapse events to back calculate the strength of pre-existing pillars using numerical modeling by estimating the stresses at which those pillars were exposed prior to the collapse [15].

**Fig. 15:**
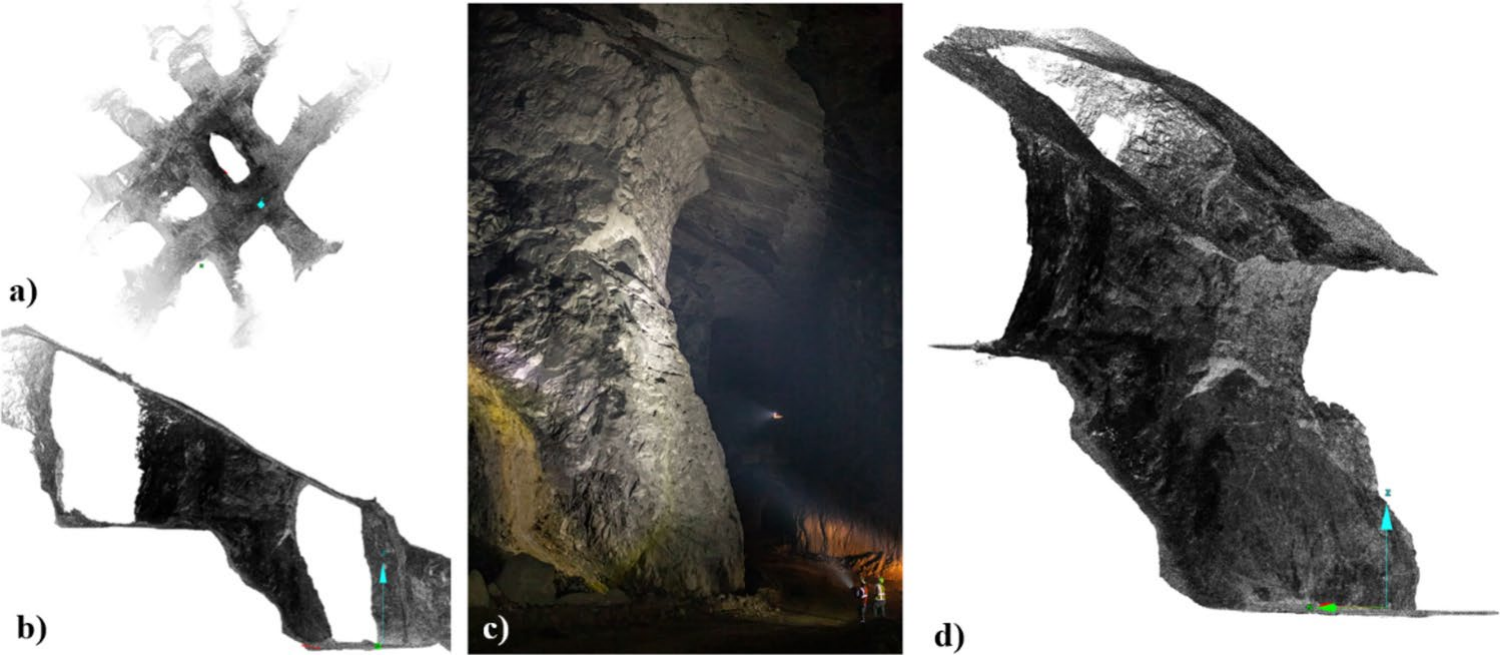
Laser scans and photos of the pillar assessed for stability. **a** Plan view, **b** cross section, **c** photography of the assessed pillar, **d** isometric view, modified after [3]

In the context of the CSM, no significant pillar failure or instability has been observed or reported. Therefore, there is not a direct way to estimate ultimate pillar strength. During the development of this project, LiDAR technologies were used to map some areas of the CSM, more specifically with the interest of mapping and characterizing discontinuities, as indicated by the authors in previous work [29, 30]. In addition, Bishop used Drone-based LiDAR and photogrammetric surveys to generate high-resolution 3D photorealistic models of some of the pillars in the same CSM (2022). Results from these surveys were proposed as a tool to compare numerical modeling results. One of the pillars in the CSM was selected and a detailed LiDAR and photogrammetric survey was performed on it. Figure 15 shows one of the selected pillar, where a) shows a plan view, b) presents a cross sectional view, c) shows one of the photos taken to this pillar at the moment of the drone based photogrammetric survey, and d) shows the final point cloud resulting from the survey. Details about the surveys and equipment used for such surveys are out of the scope of this work and are discussed in detail in [3].

**Fig. 16:**
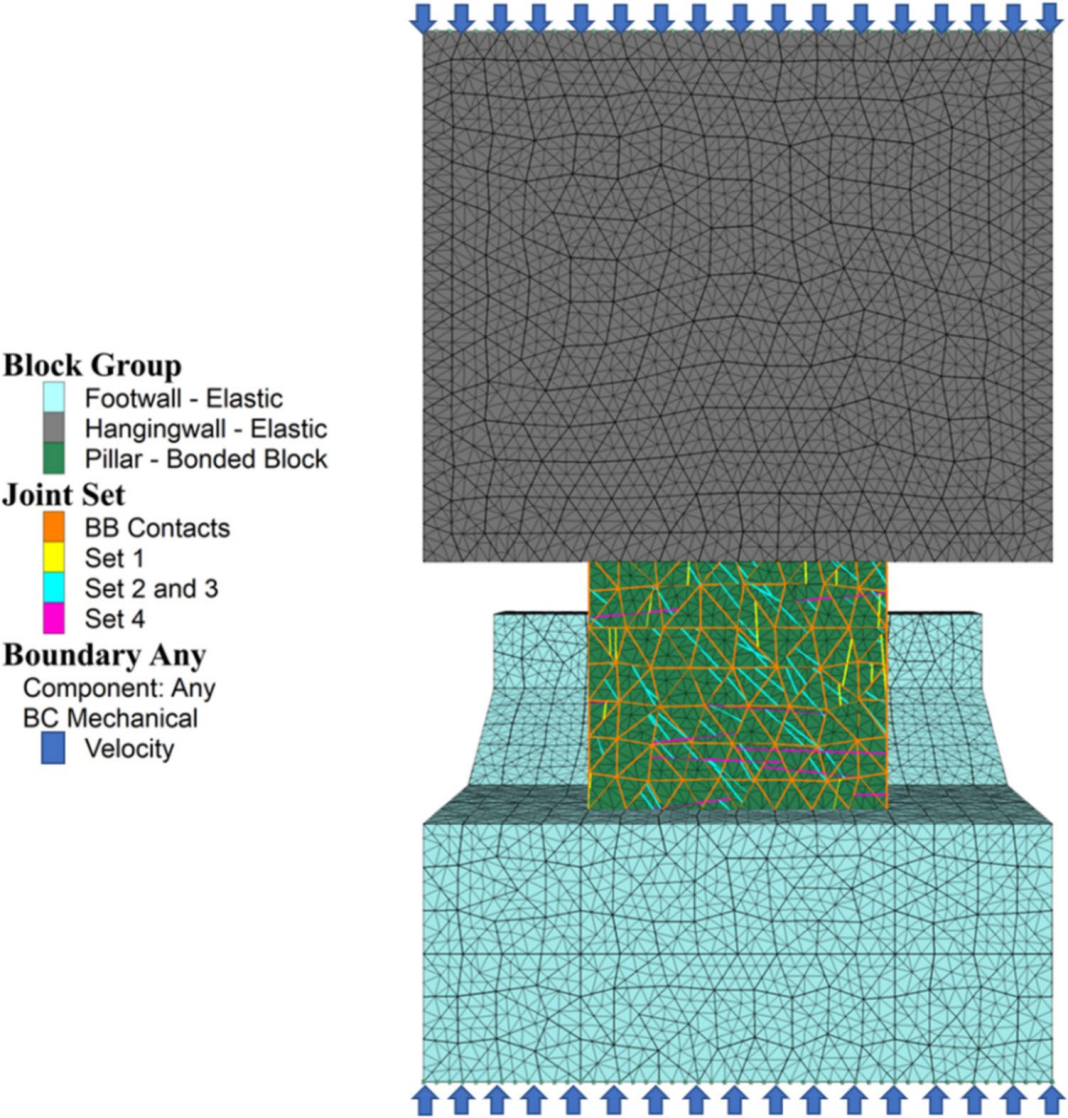
3DEC pillar stability assessment model

The scanned pillar corresponds approximately to pillar #32 in the simplified CSM model from Fig. 10. This pillar is located approximately 203 m below the actual ground surface. A static model to evaluate the response of a fractured bonded block pillar model under actual pillar loading conditions was developed in 3DEC. Figure 16 shows the pillar stability assessment model where the hangingwall and footwall blocks on the top and bottom of the pillar are modelled as elastic blocks, and the pillar intact rock material is simulated using the bonded block modeling approach. The pillar material is intersected by the filtered DFN. Discontinuity set 1 is marked in yellow, discontinuity sets 2 and 3 are marked in cyan, and discontinuity set 4 is marked in magenta. The bonded block contact faces are marked in orange.

Table 9 summarizes the properties used for each material in the model. Hangingwall and footwall materials were simulated as elastic, with a 2.7 ton/m^3^density, a 49.80 GPa deformation modulus, and a 0.22 Poisson’s ratio. The pillar was simulated using an elastic bonded block approach. The bonded blocks were generated with an approximate edge length of 1.92 m. Bonded block contact properties were defined based in the calibration properties for a 0.8 W/H ratio pillar, as reported in Table 9. For the discontinuity networks, a Mohr–Coulomb constitutive model was used with no cohesion, a 30° friction angle, a 30 GPa/m joint shear stiffness, and a 300 GPa/m joint normal stiffness.

**Table 9:** Intact Blocks and Discontinuities Properties for Pillar Stability Assessment Model

Parameter	Material
	**Pillar**
Constitutive model	**Elastic bonded blocks**
Density [ton/m3]	2.7
Young’s modulus [GPa]	64
Poisson’s ratio	0.22
BB joint normal stiffness [GPa/m]	500
BB joint shear stiffness [GPa/m]	80
BB Phi [°]	25
BB cohesion [MPa]	15.3
Tensile strength [MPa]	17
**Parameter**	**Hangingwall and footwall**
Constitutive model	Elastic
Density [ton/m3]	2.7
Young’s modulus [GPa]	49.80
Poisson’s ratio	0.19
**Parameter**	**Discontinuities**
Joint normal stiffness [GPa/m]	300
Joint shear stiffness [GPa/m]	30
Phi [°]	30
Cohesion [MPa]	0

Two loading conditions were determined for evaluating pillar performance model. For the first approach, the system was subjected to in situ stress conditions. For this case, stress scenario of a 1.5 K_o_ was used. The model was solved prior to excavation until reaching equilibrium. After in situ stresses were initialized, the material surrounding the pillar was excavated. The model was initially solved as elastic, to prevent the effects of sudden removal of the rock. Then, the material was cycled until reaching equilibrium with actual material properties. Total displacements and stress magnitudes were evaluated and compared with images and 3D models obtained from the LiDAR and photogrammetric surveys. Only a visual comparison was performed since the surveys were performed in an area where excavation had taken place long time before.

The second loading scenario was performed by applying a 0.05 m/s velocity boundary on the top and bottom of the model until pillar failure. Figure 16 indicates the velocity boundaries on the tested model. A FISH function was used to calculate vertical stress vs vertical displacement curve as the load was applied to the system. The loads were measured on the top and bottom of the hangingwall and footwall blocks by measuring reaction forces. The area on the top and bottom of the model was approximately 1785.4 m^2^, and the cross-sectional area in the middle of the pillar is 594.9 m^2^. The strength of the pillar was calculated as the strength of the system multiplied by the area of the top of the model divided by the cross-sectional area of the pillar. This analysis enabled to observe pillar failure before, during, and after ultimate pillar strength was reached.

### Stability Assessment and Validation Results

**Fig. 17:**
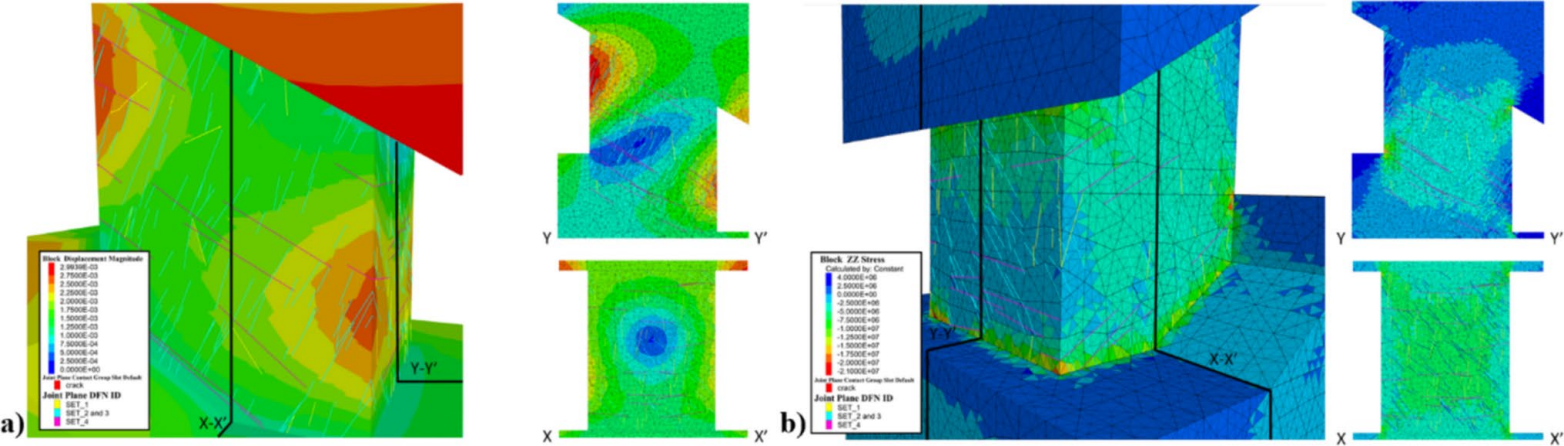
3DEC pillar stability assessment model. **a** Total displacement magnitude, **b** vertical stress distribution

Figure 17 shows the results for the initial loading condition, where pillar #32 is subjected to initial in situ stress conditions, and then surrounding material is excavated. Figure 17a highlights the displacements in the pillar surface. In addition, two cross sections across the X and Y axis of the pillars are shown. The simulation indicated maximum displacements of 2.99 mm. The figure indicates that pillar areas with larger deformations are located on the faces exposed to the mine drifts. The faces opened to the cross-cuts presented lower displacements in the order of 2 mm. It is also possible to observe that areas where discontinuities are present and intersecting each other present higher magnitudes of displacements, especially on the corners of the pillar. Figure 17b shows the pillar colored by vertical stress. In the cross section, it is possible to observe that the areas of the pillar that present less displacements are also those areas where higher stresses concentrate. The areas identified with the highest stress concentrations were the top of the pillar on the bottom stope, and the floor of the pillar on the top stope. The maximum vertical pillar stress magnitude is in the order of 21 MPa. This simulation also shows that under the assumed stress conditions, there are no bonded block contacts failing. This indicates that the intact rock material is not subjected to failure.

**Fig. 18:**
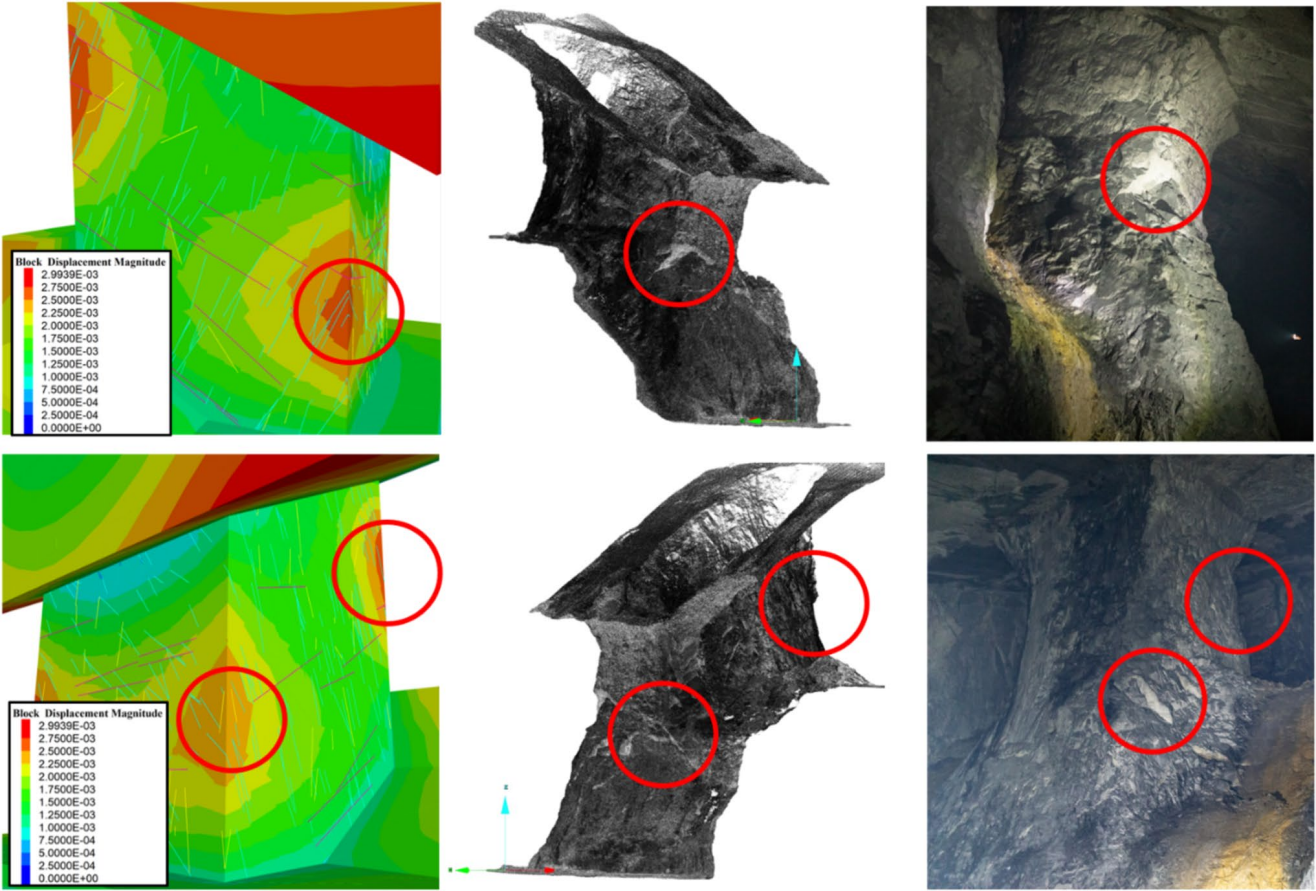
Comparison of numerical modeling results with LiDAR 3D model and pillar photos from different perspectives

Figure 18 compares the numerical modeling results with the 3D point cloud models obtained from laser scanning and photos used to generate the photorealistic models of the mine. Even though no multitemporal evaluations were performed in the CSM due to the conditions of the project, the comparison between the stochastic DEM numerical model and the 3D pillar surface shows some correlations that are worth mentioning. The figures on the left shows pillar model generated with a stochastic filtered DFN. The stochastic fracture model was able to represent some of the discontinuities that were observed in the field as it is being indicated with the red circles. The same circled areas were the areas in the numerical model that yielded higher displacements on the surface of the pillar. In the CSM, those areas presented rock material removal. The reasons for the removal of that material are not clear, since the authors were not present in the operation when the pillar of interest was excavated. However, there are two reasons that may explain the removal of material on the corners of the pillar. One explanation to this could be that the effect of blasting during the mining process loosened up the material continuous to the discontinuities causing the rock to fall during the blasting process. The other reason that could explain the removal of material on the pillar corners could be due to the mechanical scaling practices the operation performs.

**Fig. 19:**
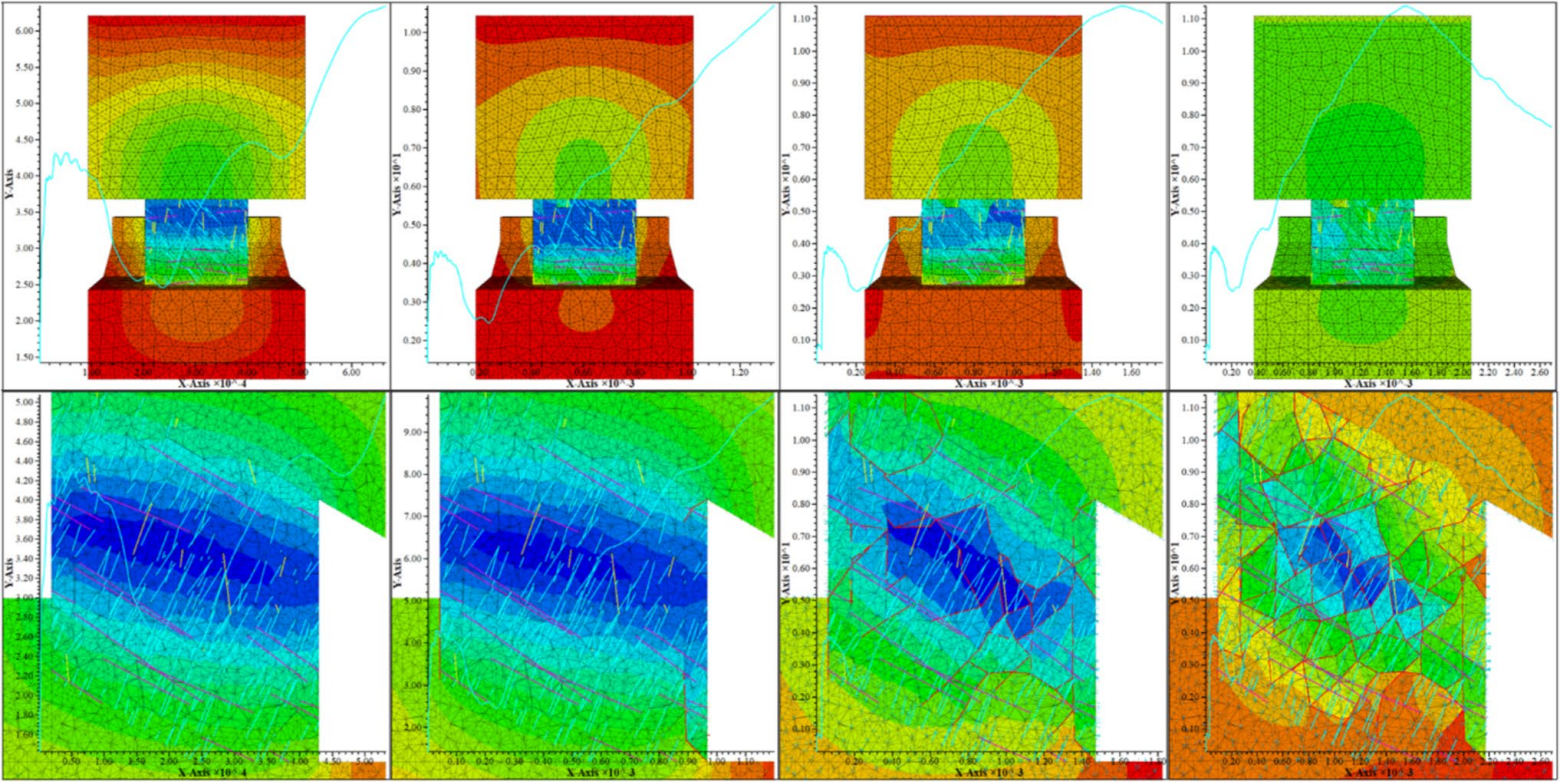
Multiple images of the fractured bonded block pillar model of pillar #32 subjected to failure load at different times during the loading process. (Top) Front view of the pillar, (bottom) cross section of the pillar in the down dip direction

**Fig. 20:**
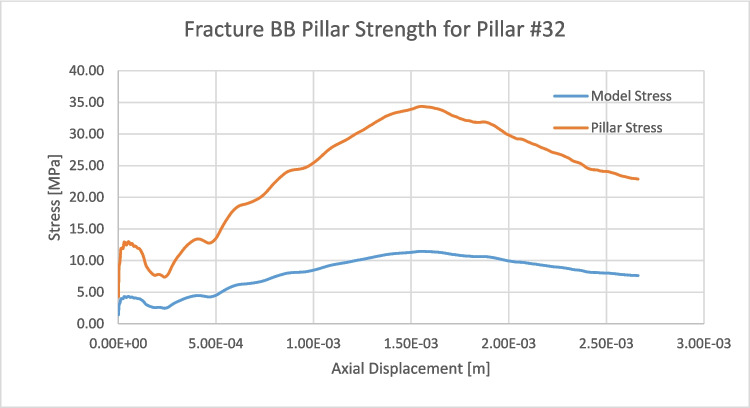
Stress-displacement curve for the fractured bonded block model for pillar #32

Figure 19 shows the results for the second loading scenario, where velocity boundaries are applied on the top and bottom of the pillar until failure. The top figures show the pillar, the hangingwall, and footwall blocks colored by displacement, and each frame from left to right indicates a different point as the stress-displacement curve evolves, starting from in situ stress condition. Stresses are being measured on the top and bottom of the model by adding up all reaction forces on the top and bottom and dividing the force by the two times the area. Figure 20 shows in blue the resulting stress-displacement curve for the average stresses measured on top and bottom of the model. The bottom images in Fig. 19 are cross sections of the pillar indicating the crack generation and propagation as the pillar reaches ultimate strength. The red lines that appear as the simulation progresses represent bonded block breakage, indicating that the intact rock material in the pillar has failed.

The force equilibrium concept was used to derive ultimate pillar strength. For this, the stress of pillar #32 was determined by multiplying the maximum model stress by the area of the top of the model divided by the cross-sectional area in the center of the pillar. All recorded stresses were divided by this aerial proportion allowing to generate a pillar stress vs displacement curve, as indicated in orange in Fig. 20. This model estimated an ultimate pillar strength of 34.4 MPa and a model ultimate strength of 11.4 MPa. The obtained pillar strength is 99.4%, the average stochastic DEM pillar strength value for a 0.8 W/H pillar, as reported in Table 5.

The pillar stability assessment on pillar #32 indicates that this pillar will be stable even if the actual stress conditions on the mine are the worst stress conditions assumed of a 1.5 horizontal to vertical stress ratio. Assuming worst stress scenario, this pillar would be subjected to an average vertical stress of 17.8 MPa, yielding a factor of safety of 1.92 assuming that the measured pillar strength in Fig. 20 is the actual pillar strength. This result agrees with the site observations and LiDAR and photogrammetric 3D pillar models, where no significant signals of pillar instability are observed. The authors suggest that a multitemporal drone-based LiDAR/photogrammetric survey as pillars are being excavated could be useful to further validate stochastic discrete element numerical models integrating DFNs and the bonded block method.

## Discussion

Results from the stochastic DEM analysis indicate that even though the presence of discontinuity sets does have an impact on pillar strength variability, their effect is minimum when compared with other parameters such as intact rock variability that has a higher COV. As it was shown, the COV for the for the NIOSH CSM pillar strength is tenfold the COV obtained for stochastic DEM pillar strength when considering discontinuity variability. These results indicate that characterizing the effect of discontinuities on pillar strength variability may not be as relevant as characterizing and having better estimates of intact rock compressive strength and its variability. The time and resources invested in running stochastic simulations to evaluate the effect of discontinuities on pillar strength variability may be reallocated to other tasks that may have greater contribution on uncertainty characterization. However, the effect of discontinuities does have to be considered in the estimation of pillar strength, since this work, just as many others authors have demonstrated, that the presence of discontinuities reduces the strength of rock pillars [6, 8, 10, 48].

Probability of failure is a term that initially may sound outrageous, just because the word failure is implicit on the term. However, this concept should be adopted and implemented as an additional tool to acknowledge the inherent changing nature of geo-materials. Designing a mine based only on factors of safety does not make the uncertainty associated with design parameters to go away. Conversely, this prevents engineers and decision makers to acknowledge that parameters such as intact rock properties, discontinuity networks strength and spatiality, stress magnitudes and orientations, and even operational conditions have variability that ultimately affects pillar performance. Elmo and Stead [7] discussed the role of behavioral factors and cognitive bias in the rock engineering practice. They discussed how these two concepts prevent the adoption of new characterization and design approaches.

International ground control best practices recommend the implementation of ground control management plans (GCMPs) as an administrative control tool to ensure the operation’s safety associated with ground instability issues [4, 9, 16, 26, 27, 35]. A GCMP may be summarized as a living document based on the application of modern risk management principles to ground control. This means that it should register and describe all the hazards related to the ground conditions, as well as identify, and assess all possible risks linked to these conditions. It should detail the expected ground conditions and acknowledge the uncertainty associated with the geology and geomechanical properties of the rock mass. These documents should also define all the engineering and operational controls required to prevent all identified ground-related risks from materializing.

An adequate GCMP is an administrative control that enables engineers and management to account for and manage ground control-related risks through the definition of “acceptable risk” levels according to each organization’s profile [43]. Design approaches and engineering controls contained in GCMPs should allow to estimate ground instability hazards’ likelihood of occurrence, so risks can be quantified according to the consequences in case one of these events occur. Hadjigeorgiou [19] summarizes a series of risk matrixes to evaluate risks in mining operations by considering event likelihood of occurance and consequences measured in different scales. If the highest probability of failure value of 0.8% obtained from this analysis is compared to common risk matrixes, the collapse of that pillar would be categorized as a rare event, the lowest qualification.

The framework proposed in this work enables to estimate pillar failure likelihood through the usage of the probability of failure in the context of pillar design and stability assessment. The applicability of this methodology was tested in a CSM, where it was shown that it provides results that may support decision-making in the stability assessment of existing pillars in an underground stone operation. Both approaches used to estimate pillar strength and pillar stresses enabled to account for the uncertainty associated with most of the design parameters such as intact rock strength, discontinuity networks variability, surface topography, rock mass elastic properties, and horizontal to vertical stress ratio. Dunn [5] discussed the importance of implementing engineering design analysis and risk assessment processes that help understanding and characterizing the uncertainty associated to mining projects. This work introduced a new approach for pillar design that helps to understand and reduce the uncertainty in the pillar design processes, ultimately improving safety in underground stone mine operations.

## Conclusions

Even though pillar collapses are events with lower probability of occurrence in comparison to other ground instability issues, the consequences of these failures are such that make these high-risk events. Therefore, design of new pillars and the stability assessment of old pillars in legacy workings should be considered from a risk management perspective. This work introduced a risk-based pillar design method that uses the reliability method to combine stochastic DEM for pillar strength estimation and stochastic continuous modeling for pillar stress determination. The proposed methodology was used to evaluate the stability of a case study underground stone mine. In addition, LiDAR and photogrammetric surveys were used to validate numerical modeling results. The following are some conclusions derived from this work:

- A fractured pillar model combining the bonded block method and a filtered DFN model was developed to estimate the effect of significant discontinuities on pillar strength. The bonded block model was calibrated with the NIOSH empirical pillar strength formula, producing results consistent with current industry standards.
- The stochastic discrete element modeling approach was used to characterize the effect of the filtered DFN on pillar strength variability. Results showed that stochastic discontinuity networks have a much smaller impact on the pillar strength coefficient of variation (COV) compared to intact rock strength variability. While discontinuities significantly influence pillar strength, their contribution to strength variability is minimal compared to intact rock properties.
- A 3D stochastic finite volume model was used to estimate stresses in a simplified CSM model. The point estimate method accounted for the variability in rock mass deformation properties on pillar stress distribution. Additionally, a parametric stress assessment considered the uncertainty in the horizontal to vertical stress ratio, enabling the estimation of mean and standard deviation values for vertical stresses on each pillar in the simplified model.
- The reliability method integrated strength and stress estimation results, enabling the estimation of a probability of failure for each pillar in a simplified CSM pillar arrangement. Since probability of failure is not a common standard for pillar design in underground stone mines, the authors proposed a threshold value of 0.05%, based on comparisons between calculated factors of safety and the accepted 1.8 factor of safety standard.
- Probability of failure should be considered an additional tool in the risk assessment process for designing, excavating, and monitoring underground mine operations. This approach accounts for the natural variability of rocks and rock masses, helping to reduce uncertainty in these materials. Implementing strategies to address and mitigate uncertainty in pillar design is crucial for improving safety in underground stone mine operations.
- A pillar stability assessment was conducted by simulating a specific pillar in the CSM using the bonded block pillar model with the filtered DFN. Results indicated the pillar was stable under current stress conditions. LiDAR and photogrammetric surveys were used to validate the numerical model, revealing correlations between the model’s performance and the observed state of the pillar. This suggests the numerical modeling approach could be extended to other sites for analyzing discontinuity-affected hard-rock pillars.

## Data Availability

Data sets generated during the current study are available from the corresponding author on reasonable request.
